# A behaviour change intervention promoting physical activity following dysvascular amputation: Protocol for a pilot study

**DOI:** 10.1371/journal.pone.0326761

**Published:** 2025-07-10

**Authors:** Crystal MacKay, Diana Zidarov, Brian Chan, Steven Dilkas, Sander L. Hitzig, Andresa R. Marinho-Buzelli, Amanda L. Mayo, Michael W. Payne, Amy Schneeberg, Julia O. Totosy de Zepetnek, Dalton Wolfe, José Zariffa, Audrey Zucker-Levin, Susan Jaglal, Brittany Pousett, Heather Underwood, William C. Miller

**Affiliations:** 1 Faculty of Health Sciences, School of Rehabilitation Therapy, Queen’s University, Kingston, Ontario, Canada; 2 Kite Research Institute, University Health Network, Toronto, Ontario, Canada; 3 Department of Physical Therapy, University of Toronto, Toronto, Ontario, Canada; 4 Centre for Interdisciplinary Research in Rehabilitation of Greater Montreal (CRIR), Montreal, Canada; 5 Institut Universitaire sur la Réadaptation en Déficience Physique de Montréal (IURDPM), Centre Intégré Universitaire de Santé et de Services Sociaux du Centre-Sud-de-l’Île-de-Montréal, Montreal, Quebec, Canada; 6 Faculty of Medicine, School of Rehabilitation, Université de Montréal, Montreal, Canada; 7 Institute of Health Policy, Management and Evaluation, University of Toronto, Toronto, Ontario, Canada; 8 West Park Healthcare Centre, University Health Network, Toronto, Ontario, Canada; 9 Division of Physical Medicine and Rehabilitation, Temerty Faculty of Medicine, University of Toronto, Toronto, Ontario, Canada; 10 St. John’s Rehab Research Program, Sunnybrook Research Institute, Sunnybrook Health Sciences Centre, Toronto, Ontario, Canada; 11 Department of Occupational Science and Occupational Therapy, Temerty Faculty of Medicine, University of Toronto, Toronto, Ontario, Canada; 12 Department of Physical Medicine and Rehabilitation, Schulich School of Medicine and Dentistry, Western University, London, Ontario, Canada; 13 University of British Columbia, Vancouver, British Columbia, Canada; 14 Faculty of Kinesiology and Health Studies, University of Regina, Regina, Saskatchewan, Canada; 15 Lawson Health Research Institute, London, Ontario, Canada; 16 Faculty of Health Science, University of Western Ontario, London, Ontario, Canada; 17 Institute of Biomedical Engineering, University of Toronto, Toronto, Ontario, Canada; 18 School of Rehabilitation Science, University of Saskatchewan, Saskatoon, Saskatchewan, Canada; 19 GF Strong Rehabilitation, Vancouver, British Columbia, Canada; 20 Occupational Science and Occupational Therapy, University of British Columbia, Vancouver, British Columbia, Canada; PLOS: Public Library of Science, UNITED KINGDOM OF GREAT BRITAIN AND NORTHERN IRELAND

## Abstract

**Background:**

Diabetes-related lower limb amputation (LLA) is a leading cause of disability globally, impacting individuals’ physical and mental health, and ultimately their quality of life. Physical activity can reduce risk of chronic disease and mortality while improving quality of life. However, people with LLA often have reduced balance and walking ability resulting in sedentary behaviour. We co-created a physical activity intervention, **IM**proving **P**hysical **A**ctivity through **C**oaching and **T**echnology following **L**ower **L**imb **L**oss (IMPACT-L3), to support physical activity behaviour change in people with dysvasular LLA. To date, no studies have assessed a peer-led physical activity behaviour change intervention for people with LLA. Prior to launching a large trial, a pilot study is required to assess feasibility and optimize design of a future trial.

**Methods:**

This pilot study is a parallel group randomized controlled trial (RCT) with an embedded qualitative component. The intervention group will have access to once-weekly virtual peer coaching sessions with a peer trained in brief action planning; web-based physical activity modules; and a wearable activity monitor for 8 weeks. The control group will continue usual care and be offered the intervention at the end of the follow-up period. Data on feasibility will be collected including assessment of process, resource, management and treatment indicators. The proposed primary outcomes will be measured at baseline, post-intervention and one month later: total physical activity counts per day measured by the ActiGraphTM activity monitor and self-efficacy measured by the Self-efficacy for Exercise scale. Secondary measures include patient reported outcome measures of physical activity, mobility, depression, social participation, balance confidence and quality of life. Semi-structured interviews will explore feasibility and acceptability of the intervention to participants and peers.

**Discussion:**

This study will inform the design of a definitive RCT to determine the effectiveness of a peer-led physical activity intervention for people with dysvascular LLA.

## Introduction

Lower limb amputation (LLA) is a significant life event which impacts mobility, health, and ultimately quality of life (QoL) [1–6]. In the United States alone, 185,000 LLAs are performed annually, with healthcare costs estimated at> $4.3 billion [7]. The number of LLAs are projected to double by 2050 as a result of increasing rates of diabetes and the aging population [8]. In Canada, 7,300 people have a LLA each year [9]; more than 80% of these amputations result from complications of diabetes and/or peripheral vascular diseases (dysvascular LLAs) [9]. In addition to disability resulting from LLA, individuals with dysvascular LLA often have multimorbidity (mean of five health conditions) [10,11], and 37% of individuals require a contralateral or revision amputation within five years, which can further impact function and quality of life [12]. Moreover, cardiovascular comorbidities are cited as the leading cause of death in people with dysvascular LLA [13–20]. Despite these complex health challenges, rehabilitation services for people with LLA are often limited [21,22]. In Canada, only 36% of adults with LLA receive inpatient rehabilitation and services are more available in urban than remote areas [23]. Taken together, these findings underscore the need for accessible, low-cost approaches to optimize health, social participation and QoL in this population.

There is strong evidence that physical activity (PA) reduces risk of chronic disease, all-cause and cardiovascular mortality and improves QoL [24]. Exercise is a cornerstone of management of LLA [25] and PA interventions may confer additional benefits for people with dysvascular LLA such as improving diabetes outcomes and prevention and management of cardiovascular disease [24,26]. Despite the potential benefits, people with LLA have low PA levels [27–30]. People with dysvascular LLA have reduced strength, balance, cardiorespiratory fitness, walking ability and increased energy expenditure during ambulation [31–36]. Consequently, they have impaired mobility, which may lead to sedentary behaviour. For instance, across studies examining physical activity in people with dysvascular LLA, step counts ranged from 1250 steps/day in older prosthesis users to 3809 ± 2189 steps/day in people with diabetes-related LLA [27–30]. In one study, people with dysvascular LLA accumulated 24 ± 41 minutes per week of moderate to vigorous physical activity, well below the recommended 150 minutes per week [37]. Importantly, not everyone with LLA can use a prosthesis due to muscle weakness, cognitive impairment or skin problems [38,39], and it can be difficult for wheelchair users to accrue adequate physical activity [40]. Other known barriers to physical activity following LLA include lack of knowledge and self-efficacy, transportation to get to spaces to participate in physical activity, geographic features of the environment (e.g., stairs), and availability of physical activity programs for people with LLA [41,42].

To date, research has focused on studying the effects of combined exercise training programs for people with LLA [43]. A systematic review found that exercise performed one to three times per week improved balance, walking speed, walking endurance and transfer ability in adults with LLA, especially when combining aerobic exercises with lower limb strengthening or balance exercises [44]. However, few individuals with dysvascular LLA were included in the studies (35%) [44]. Of note, only two studies were found that examined biobehavioral interventions to support physical activity behaviour change. In a randomized single blind feasibility trial of a 12-week telehealth intervention delivered by therapists for people with non-traumatic LLA (1–5 years following amputation), the findings established the feasibility of the intervention including a 90% retention rate, low safety risk and high acceptability [45]. In another therapist-delivered remote intervention promoting exercise and self-management for people within the first six months post dysvascular amputation, daily step counts increased to 1135 steps per day in the intervention group compared to 144 steps per day in the control group after 12 weeks (p = .03) [46]. While these studies had small sample sizes with predominantly male participants, findings suggest that eHealth behaviour change interventions may be a promising approach to optimize physical activity for this population.

Current evidence suggests that multifaceted interventions that incorporate social support and behaviour change techniques increase physical activity in older adults and people with chronic illness [47–50]. Individuals with LLA have identified support from others to engage in physical activity as important, including follow-up phone calls and counselling [42]. In particular, social support from peers with limb loss was perceived as a facilitator of physical activity [51]. Peer involvement in non-amputee physical activity programs has been shown to be a promising approach to improve physical activity levels [52,53]. A meta-analysis of 17 studies which examined the effects of peer-led self-management programs on physical activity showed a moderate pooled effect size (standardized mean difference = 0.4, p < 0.001) and interventions were shown to be safe with high adherence [54].

In a national meeting in Canada, stakeholders ranked research on peer led physical activity programs for people with dysvascular LLA as a top research priority [55]. To date, no peer-led physical activity interventions for people with dysvascular LLA have been evaluated [43,56] and few studies have used technology to optimize physical activity in this population. To address this gap, we co-created a novel physical activity behaviour change intervention comprised of peer coaching, wearable technology and education modules (**IM**proving **P**hysical **A**ctivity through **C**oaching and **T**echnology following **L**ower **L**imb **L**oss (**IMPACT-L3**)). Prior to conducting a definitive trial to determine effectiveness of IMPACT-L3, a pilot study is required to assess feasibility and optimize design of a future trial. Our aim is to assess the feasibility of conducting a definitive randomized controlled trial (RCT) to determine the effectiveness of a virtual peer-led physical activity intervention on levels of physical activity and self-efficacy in people with dysvascular LLA. Our specific objectives are to:

1) Evaluate feasibility according to indicators of process, resources, management and treatment.
2) Explore perceptions of program characteristics, program implementation and study procedures among individuals with LLA; and
3) Explore the perceived feasibility and acceptability of the program among peer coaches.
4) Inform a sample size calculation for a future trial of effectiveness.

## Materials and methods

### Study design

**Fig 1:**
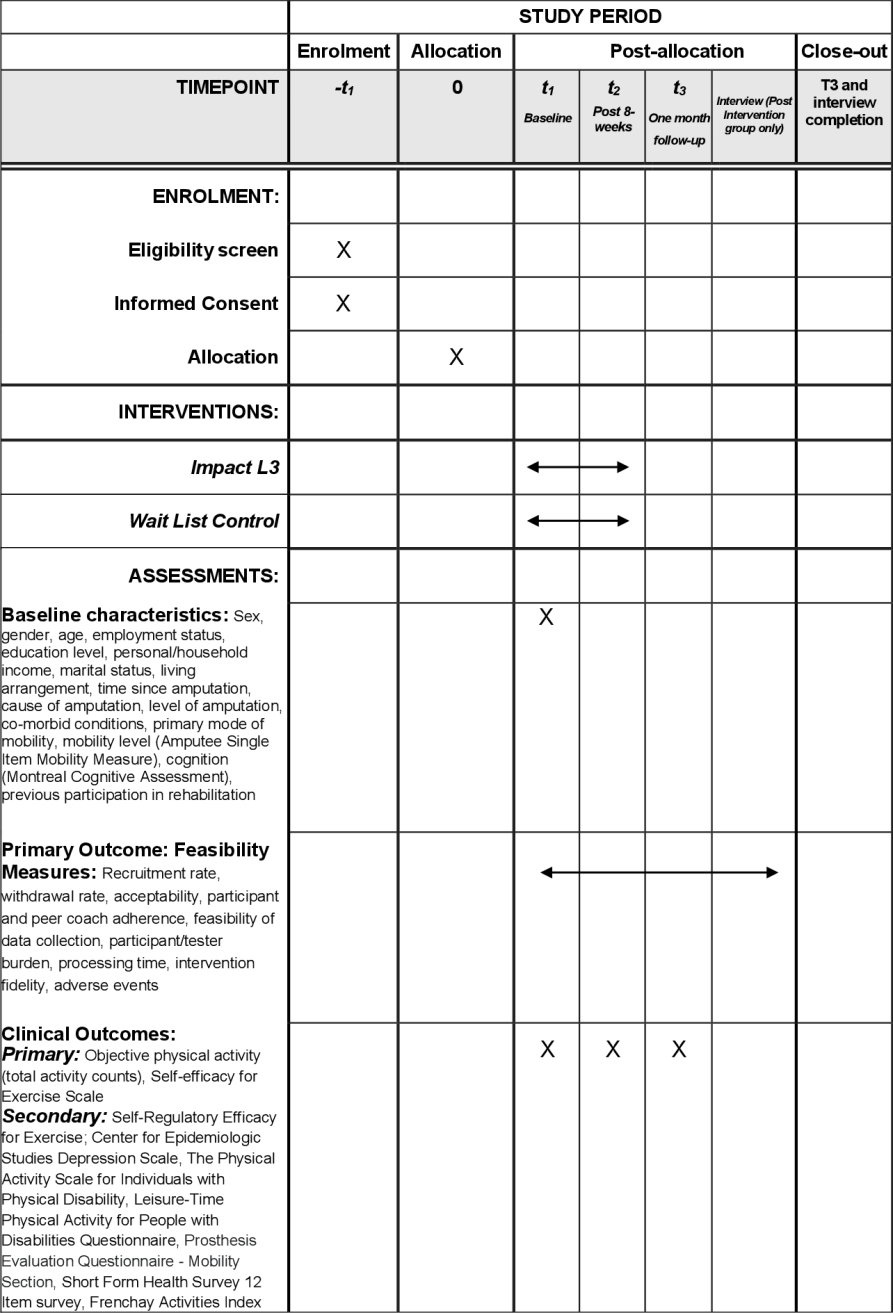
Schedule of enrolment, interventions, and assessments.

This pilot study is a parallel group randomized controlled trial (RCT) with a nested qualitative component. A pilot study is a subset of feasibility studies which asks questions about feasibility (whether the future trial can be done and, if so, how) but with a key design feature: in the pilot study, the future RCT is conducted on a smaller scale [57]. Prospective recruitment, concealed group allocation, evaluator masked outcome evaluation and waitlist control will be employed. The research is guided by the Medical Research Council (MRC) framework for evaluating complex interventions [58]. The trial will be conducted and reported following the Consolidation Standards of Reporting Trials statement, with consideration for pilot and feasibility studies as recommended by Thabane et Lancaster [59]. We used the Standard Protocol Items: Recommendations for Interventional Trials (SPIRIT) to report the study protocol (please see S1 Checklist) [60]. Fig 1 presents the SPIRIT schedule of enrollment, interventions, and assessments.

### Setting

All components of the intervention and study protocol will be delivered remotely. This study will be conducted with community-dwelling adults with dysvascular LLA living in Canada.

### Recruitment

A multi-pronged recruitment strategy will be used. Participants will be recruited from rehabilitation sites by research staff and/or clinical collaborators as patients who have completed rehabilitation services as an inpatient or outpatient following LLA often continue to have long term follow-up appointments with the clinical team (e.g., annual visits, assessment of prosthesis, etc.). This strategy will be used because: 1) it enables access to a large population of individuals with LLA to enhance recruitment; 2) and the characteristics of the population are representative of Canadians with LLA (e.g., primarily older individuals with dysvascular LLA. Potential participants will self-identify or be identified by a member of the research team or health professional. Interested individuals can contact the study team directly or provide consent to be contacted by a researcher.

Study posters and advertisements will be used to recruit potential participants at rehabilitation sites/clinics and community organizations. Study advertisements will be distributed using social media platforms and websites of amputation organizations across Canada. Potentially eligible participants will be asked to contact the research team by email or phone using details provided on recruitment materials. A researcher will then contact interested individuals by email and telephone to screen for eligibility.

### Participants

Individuals who meet the following inclusion criteria will be included: 1) at least one dysvascular LLA (LLA due to diabetes or vascular disease); 2) major LLA (at the ankle or above); 3) living in the community; 4) adult at least 18 years of age; 5) comfortable communicating in English and able to understand basic English; and 6) receptive to using a phone or tablet (e.g., to enable peer coaching, access to modules.) Participants may be ambulatory with a prosthesis and/or use a wheelchair.

Exclusion criteria include: 1) actively receiving rehabilitation services related to physical activity/mobility; 2) recommended medical supervision for physical activity by health care provider, or skin problems preventing usual activity and 3) not able to provide informed consent.

Participants who are interested in taking part in the study but who do not have access to a device will be offered the use of a device to borrow.

### Trial intervention: IMPACT-L3

Participants allocated to the intervention group will receive IMPACT-L3, a physical activity behaviour change intervention which was co-designed using findings from our qualitative research [51,61] and prior literature and theory. Two theories provide a framework for IMPACT-L3. One is social cognitive theory, which is a useful theoretical lens for incorporating self-efficacy into interventions [62,63]. Self-efficacy is informed by skill mastery, vicarious experience, verbal persuasion, and reinterpretation of physiological and affective symptoms [64]. The other is self-determination theory, which provides a framework for cultivating an autonomy-supportive social environment that promotes behavior change [65]. This is achieved by satisfying three basic psychological needs of autonomy (i.e., volition in one’s own behavior), competence (i.e., interacting effectively with one’s environment by mastering tasks), and relatedness (i.e., sense of belonging) [65].

IMPACT-L3 is comprised of these 3 components:

Peer health coaching (~30 minutes weekly) will be delivered by a peer trainer over 8 weeks. Peer trainers, who have experienced a dysvascular LLA themselves, will be matched to a participant based on gender and/or level of amputation to promote a sense of understanding and belonging. Individuals with higher level amputations (i.e., above knee) are less physically active and face more challenges with mobility (e.g., greater energy expenditure) [28,66], and may benefit from a peer with similar experience. Peers will be trained to implement the intervention including specialized training in brief action planning (BAP), a structured support technique grounded in the principals and practices of motivational interviewing. Training will be delivered by an organization which provides certified training in BAP (Centre for Collaboration, Motivation and Innovation) [67] including an online course as well as practice and feedback. The trained peer will help participants set goals and create an action plan for physical activity that they feel confident that they can achieve. During weekly meetings, peers will help participants problem solve challenges to physical activity and overcome barriers. The peer trainer will deliver the intervention through voice or video calls (depending on preference). Video calls will be preferred because “face-to-face” interactions may reinforce vicarious experiences (e.g., peers demonstrate movements). Peers will complete a standardized form at every interaction to review goals, goal progression, action plans, and document barriers/facilitators. Weekly debriefings among peers and the research team will problem solve challenges and monitor peer burden.

Education Modules: To improve competence and skills to enhance physical activity, participants will have access to five web-based modules developed based on qualitative interviews and co-design workshops with people with LLA. Modules include content on PA including benefits of PA, types of PA (strengthening, aerobic, balance, flexibility), intensity of activity (light, moderate, vigorous), exercise safety and limb management to enable PA, and recommended PA. The modules include videos demonstrating these activities. Participants will be given a login and will be asked to review modules at their own pace (one per week) for the first 5 weeks.

Activity Tracker: To support behaviour change, participants will be provided with a wearable to track their physical activity: an Apple Watch to be worn at the wrist of the non-dominant side. An off-the-shelf wearable was selected for the study to optimize sustainability of the intervention in a real-world setting. The Apple Watch has been shown to be accurate in tracking step count in a range of populations [68] and can measure wheelchair pushing thus making it the best option available [69]. Participants will be trained to wear and use the Apple Watch 24 hours a day for the full 8 week duration of the study, including water-based activity. Wearables that provide personalized, and actionable feedback promote better behavior change outcomes [70]. Data from the Apple Watch will be shared verbally with the peer during discussions between participants and peers during the weekly virtual sessions to facilitate behaviour change (e.g., inform goal setting).

### Control intervention

The control group will continue with their usual health care and be offered the intervention program at the end of the follow-up period (wait-list control).

### Measurements

#### Feasibility outcomes.

The primary outcome of this study is feasibility of implementing the intervention and conducting the trial. Feasibility indicators will be collected including assessment of process, resource, management and treatment indicators.

**Process indicators:** *Recruitment rate* will be evaluated by the number and proportion of participants recruited in total and the number of peers recruited in total. *Withdrawal rate* will be calculated as the percent of study participants withdrawing by week 9 (T2) and 3 months (T3). *Acceptability* will be assessed in qualitative interviews and by the theoretical framework of acceptability (TFA) [71], a brief questionnaire developed to assess acceptability in the design, evaluation and implementation of interventions.

**Resource indicators:** *Participant adherence* will be measured as the percentage of peer coaching sessions participants attend. Participants’ usage of web-based modules will be measured (i.e., number of completed modules, number of logins). *Peer coach adherence* will be assessed by tracking the total number of peer coaching sessions attended by the peer-trainer. *Participant and tester burden* will be measured by the amount of time it took to administer study outcomes at T1, T2, and T3 and the acceptability of the evaluation time commitment from the perspective of participants. *Feasibility of data collection* will be evaluated as the percentage of participants with complete data on each measure at each evaluation time point. For accelerometers, the percentage of devices that were returned at baseline and follow-up (T2 and T3) and the amount of valid wear time will be assessed.

**Management indicators:** *Participant processing time* will be assessed as time from initial contact to enrolment. *Intervention fidelity* will focus on adherent and competent delivery of the intervention. It will be evaluated using the study-specific checklist outlining key components of the intervention completed by peers. A subset of peer coaching sessions will be recorded and reviewed by research staff using the checklist.

**Treatment indicators:** *Adverse events* will be measured as the number of adverse events that occurred during physical activity for the intervention. Adverse events (e.g., falls) will be documented by peers on the standardized form used at each coaching session.

#### Clinical outcomes.

**Primary outcomes for full trial:** ***Objective Physical Activity (Accelerometer)*.** A lightweight tri-axial accelerometer (ActiGraph™ wGT3X-BT) will be used to measure total activity counts. The total volume of physical activity (activity counts) will be measured. This measure has the advantage of integrating the frequency, intensity, and duration of movement and combining them into an overall measure of PA [72]. The sum of the total count for the day will be used and averaged over the measurement period. Total activity counts can be a better metric than the number of minutes per day spent in various physical activity intensity categories because it incorporates all levels of intensity. Recent evidence suggests that light, moderate and vigorous activity **all** have health benefits [73,74]. Ambulatory participants will wear the tri-axial accelerometer on a waist belt on the side of the shortest residual limb which provides the most valid data in people with LLA [75]. Wheelchair users will wear an ActiGraph™ wGT3X-BT on the non-dominant arm [76]. For wheelchair users a second ActiGraph™ may be placed on the rear wheel (waterproof box installed on the rear wheel using tie wraps). Participants will wear the accelerometer at all times except while bathing or swimming for a period of 7 days pre- and post-intervention and at 1-month follow-up. Only data from days in which participants wear the activity monitors for >10 hours per day will be included in the analyses as per previous approaches, including individuals with dysvascular amputation [46].

***Self-efficacy for exercise scale*.** A self-report measure (9 items) that includes situations that may influence physical activity participation. Participants will respond to each item on a 0 (not very confident) to 10 (very confident) scale. This is a valid measure of exercise self-efficacy in older adults [77].

***Secondary outcomes: Self-Efficacy for Exercise – Dysvascular Lower Limb Amputees*.** A four-item measure will be administered in addition the standardized self-efficacy for exercise measure and self-regulatory efficacy for exercise measure to address contextual factors relevant to dysvascular LLA. Items are informed by a meta synthesis of data from individuals with dysvascular lower limb amputations as well as recommendations from the literature for measuring self-efficacy [42]. These items represent physical activity challenges reported by this subgroup.

***Self-Regulatory Efficacy for Exercise*.** An eight-item measure to assess participants’ confidence in their ability to manage their exercise. The measure pertains to behaviours necessary to self-regulate exercise over the next 4 weeks, such as scheduling exercise, planning exercise sessions, overcoming barriers that may interfere with exercise, and preventing relapse by overcoming temporary exercise lapses. Items are assessed using a confidence scale ranging from 0 per cent (not at all confident) to 100 per cent (completely confident) and in accordance with recommendations in the literature [78,79].These items have been used previously in exercise research, in which internal consistencies ranged from 0.84 to 0.93 (e.g., Woodgate and Brawley [79]).

***Center for Epidemiologic Studies Depression Scale (CES-D)*.** The CES-D will assess participant’s self-rated depressive symptoms. It is a 20-item scale that refers to symptoms in the last week. The sum of the 20 items provides a score ranging from 0–60 with higher scores indicating greater symptoms. The CES-D shows good internal consistency (Cronbach’s alpha coefficient of 0.85) [80]. The CES-D is the best supported measure for diabetes [80] and has been used in RCTs with patients with LLA.

***The Physical Activity Scale for Individuals with Physical Disability (PASIPD)*.** The PASIPD is a 13-item questionnaire with subscales measuring leisure time, household and work-related activities [81]. The PASIPD has demonstrated reliability and validity in people with physical disabilities [82].

***Leisure-Time Physical Activity for People with Disabilities Questionnaire (LTPAQ-D)*.** The LTPAQ-D is a self-report measure that assesses minutes of mild-, moderate-, and vigorous-intensity leisure time PA (i.e., activity that requires physical exertion and that one chooses to do in their free time performed over the past 7 days. Support for the LTPAQ’s criterion validity and test–retest reliability has been demonstrated in people with spinal cord injury and other disabilities [83].

***Activities-specific Balance Confidence scale (ABC)*.** The ABC is a self-report measure used to assess perceived balance confidence. The total score varies from 0 to 100, with higher scores indicating more confidence. Validity and test-retest reliability (ICC = 0.91) have been shown for people with LLAs [84].

***Prosthesis Evaluation Questionnaire – Mobility Section (PEQ-MS)***. The PEQ-MS score will be used to measure the amount of difficulty completing locomotion tasks. The PEQ-MS is a reliable (ICC: 0.0.73–0.90) and internally consistent (Cronbach alpha = 0.96) measure in people with LLA [85,86].

***Short Form Health Survey 12 Item survey (SF-12)*.** The SF-12 will be used to measure health-related QoL. Twelve items are categorized into a physical and a mental domain; the scores of the QoL domains range from 0 to 100, with higher scores referring to higher quality of life. Several studies have reported good psychometric properties of the SF-12 including studies in older adults [87].

***Frenchay Activities Index (FAI)*.** The 15-item FAI captures information on social activities. The items reflect the frequency of performance of basic and instrumental activities of daily living over three domains. The total scores range from 0 (very limited) to 45 (very active). Research demonstrates that this index is reliable and valid for use in individuals with LLA [88].

#### Sociodemographic and clinical characteristics.

Demographic characteristics will be collected by questionnaire. Participants will be asked about sex at birth (male, female or intersex), and what best describes their current gender identity (woman, man, non-binary etc.). Age, employment status, education level, personal/household income, marital status, and living arrangement will also be collected. LLA characteristics will be collected: time since amputation, cause of amputation, level of amputation, co-morbid conditions, primary mode of mobility, mobility level (measured by the Amputee Single Item Mobility Measure [89]), cognition (measured by the Montreal Cognitive Assessment [90]) and previous participation in rehabilitation. Self-reported healthcare use during the intervention will be documented.

**Fig 2:**
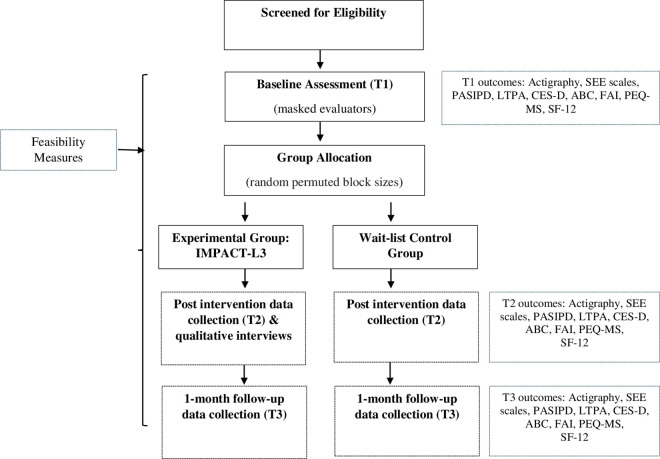
Study flow chart.

**Timeline:** Data will be collected at baseline (T1), within one-week post-intervention (T2) to examine the immediate post-intervention effects, and 1 month follow-up (T3) to see if effects are maintained. The one-on-one semi-structured interviews will be conducted following the intervention with the IMPACT-L3 group to explore perceptions of the protocol and intervention. Fig 2 shows the flow diagram of the intervention. All data collection will occur online via Zoom, Teams or WebEx utilizing a secure connection.

Recruitment commenced on January 2, 2025 (enrollment on January 23, 2025) and is anticipated to be completed on December 31, 2025 with data collection completed by March 31, 2026. Results are expected by December 31, 2026.

**Participant incentives:** Participants will receive a gift certificate valued at $50 for each of the 3 assessments. Participants and peer coaches will be asked to take part in a qualitative interview. All those who complete an interview will receive a gift certificate valued at $50.

**Sample size:** Since this is a pilot study, a formal sample size calculation was not performed. The recruitment of 20 participants per arm of the trial is judged to be feasible and will produce a robust amount of data (allowing a 20% drop out). This is consistent with Whitehead et. al.’s suggestions that pilot trial sample sizes for each treatment arm should be 15 for standardized effect sizes that are medium (0.5) [91].

**Sequence generation and randomization:** We will use a central computerized randomization process. Randomization with permuted block randomization of varying block size (2 and 4) will be conducted. The allocation schedule will be created using an online tool by a statistician who is not involved in recruitment.

A research coordinator will screen potential participants. Consent Forms will be reviewed, signed and returned online via REDcap (REDCap Software, Vanderbilt University and National Institute of Health, USA). Individuals who are not able to sign the Consent Form via REDCap will be given an option to sign and return the Consent Form in person, via email or by mail with a pre-stamped and addressed envelope. After obtaining informed consent, the research coordinator will schedule a meeting with a masked assessor to complete the baseline assessment. An independent statistician will provide the randomization list. Upon enrolment, a study ID number will be allocated to each participant. After completing the baseline assessment, the research coordinator will reveal the group allocation of the participant.

### Masking

Due to the nature of the intervention, masking to receipt (or not) of the intervention is impossible. The assessor will be masked to group allocation. We will ask participants not to disclose their group allocation during assessments.

### Data collection methods

All participants will be asked to complete the online questionnaires described above via REDCap. Participants will be provided with a link by email. Individuals who are not able to complete the questionnaires via REDCap will be given an option to receive the questionnaire via email or by mail. To increase response rates, a research coordinator will book a time with the participant to support completion of the questionnaires (the participant will self-complete the questionnaire in REDCap; the research staff will be available to support any technical challenges). The ActiGraphTM activity monitor will be mailed to the participant with instructions on how to use it to measure total activity counts per day. A research coordinator will be available to support the participant via phone or Zoom.

### Retention

A research coordinator will contact all participants every four weeks until the end of study to remind them of the next assessment session and confirm the schedule.

### Data management

A research coordinator will screen potential participants, obtain informed consent, and schedule assessments. All data will be collected by a trained evaluator who will be masked to group allocation. A research coordinator with experience in qualitative research will be trained to complete the qualitative interviews. Quantitative and qualitative data will be password protected and stored in a secure server with necessary firewalls and security measures in place, hosted by the primary hospital. Hard copy data will be stored securely at primary hospital. Only members of the immediate research team will have access to the data. Participants will be assigned a participant ID number when they enroll into the study to maintain participant anonymity.

### Data analysis

#### Feasibility measures.

Descriptive statistics (mean, standard deviation, counts (percentage)) will be used to summarize continuous and categorical data, as appropriate.

#### Clinical measures.

As a pilot study, the analysis will be mainly descriptive. We will use the data from this pilot study summarized overall and by group to help inform outcomes measures and sample size estimation for the main trial. The baseline activity, health and demographic characteristics and all other covariates for the intervention and control groups will be described by frequencies and percentages and mean and standard deviation as appropriate. The proposed primary and secondary outcomes will be described by mean, median, standard deviation and interquartile range. They will be visualized with box and whisker plots and histograms by intervention group and time point. Descriptive statistics and estimation, using confidence intervals, will be used to inform sample size estimation. The confidence interval will be interpreted with consideration of clinical relevance. If sufficient data, the relationship between intervention/control and the primary and secondary outcomes will be examined using linear mixed effects models including participant ID as a random effect and the observations taken at T2 and T3 as the outcome while controlling for the baseline observation.

### Progression criteria to a definitive trial

Mellor et al. (2023) [92] recommend using guidelines rather than rules when using progression criteria in a feasibility study, avoiding consideration of one indicator in isolation, and involvement of partners in the decision making. These principles, with the RAG (red amber green) or traffic light approach, will be used to guide the research team in determining what changes may be required to address identified challenges based on the indicators and in determining the feasibility of progression to a trial. This traffic light system highlights problems that have been faced in the pilot trial, with red indicating major problems that require urgent attention (and perhaps cannot be remedied), amber indicating minor problems that require attention, and green indicating areas of no concern. See Table 1 for criteria.

**Table 1:** Feasibility indicators and measurement criteria.

Feasibility Component	Indicator	Go: Proceed with RCT	Amend: Amendments to proceed	Stop: Consider whether RCT can proceed and only proceed if changes are possible
***Process***				
Recruitment rate	Number and proportion of participants recruited and the number of peers recruited	Recruitment of 40 participants within 12 months and recruitment of the targeted number of peers	If targets reached but it takes longer than predicted or >80% of sample recruited	Recruit <60% of sample
Withdrawal rate	Percent of study participants withdrawing by T2 and T3	Withdrawal of ≤ 20% of participants AT T2 and T3 evaluations	Withdrawal of 21–40% of participants at T2 and T3 evaluations	Withdrawal of >40% of participants at T2 and T3 evaluations
Acceptability	-Theoretical framework of acceptability (TFA) -Qualitative interviews	General acceptability mean score ≥4 in TFA Qualitative interviews suggest the intervention is highly acceptable	Qualitative interviews suggest the intervention is acceptable	Qualitative interviews suggest the intervention is possibly acceptable
***Resources***				
Participant adherence	Percentage of peer coaching sessions participants attend. Participants’ usage of web-based modules will be measured (i.e., completed modules, logins).	Participant attendance at >85% of peer coaching sessions	Participant attendance at 60–85% of peer coaching sessions	Participant attendance at <60% of peer coaching sessions
Peer coach adherence	Number of peer coaching sessions attended by the peer- trainer	Peer attendance at >90% of peer coaching sessions	Peer attendance at 60–90% of peer coaching sessions	Peer attendance at <60% of peer coaching sessions
Feasibility of data collection	Percentage of participants with complete data at each evaluation time point. For accelerometers, the percentage of devices that were returned at T2 and T3 and the amount of valid wear time.	>90% complete data on baseline evaluations and >80% for T2 and T3 evaluations	70-90% complete data on baseline evaluations and 60-80% for T2 and T3 evaluations	<70% complete data on baseline evaluations <60% complete data on T2 and T3 evaluations
Participant & Tester burden	-Amount of time it took to administer study outcomes at T1, T2, and T3 -Perceived tester burden (interviews)	> 85% of participants complete in ≤ 2 h	70-85% of participants complete in ≤ 2 h	<70% of participants complete in ≤ 2 h
***Management***				
Participant processing time	Time from initial contact to enrolment	Mean time is < 10 days	Mean time is 10–15 days	Mean time is > 15 days
Intervention fidelity	-Study-specific checklist -A subset of peer coaching sessions will be reviewed by research staff using the checklist	> 90% of checklist completed	70-90% of checklist completed	<70% of checklist completed
***Treatment***				
Adverse events	Number of adverse events that occurred during the IMPACT-L3 intervention.	No major injuries or adverse events reported	At least one major injuries or adverse events reported	>1 major injuries or adverse events reported

### Nested qualitative study

We will use a qualitative descriptive approach [93] to understand participants’ and peers’ experiences with IMPACT-L3. This component will be critical to improving the intervention and refining the protocol for the definitive trial. The research will be situated within an interpretive research paradigm [94]. To explore perceptions of recruitment approaches, data collection procedures and measures, and acceptability of program implementation (Objective 2), one-on-one semi-structured telephone or Zoom interviews lasting ~45–60 minutes will be conducted with participants after completion of the intervention (week 9). Participants will be asked to share their perceptions of program characteristics. For example, participants will be asked about their perceptions of using Apple Watch to support monitoring an feedback of physical activity including the appropriateness of the data provided (e.g., distance walked/wheeled) and the usability of the device. Findings will inform refinements of the intervention, if needed. Data will be collected until data saturation. Based on prior research [42], we anticipate requiring all 20 participants from the intervention group, which should ensure variation in age, gender and level of amputation.

For objective 3, we will conduct semi-structured interviews with all consenting peers to understand their experiences and identify considerations for optimizing the intervention. Inductive thematic analysis will be employed to analyze qualitative interviews [95]. NVivo software will be used for qualitative analysis. Team members will independently code 2–3 transcripts to develop a preliminary coding framework. The coding framework will be applied to four transcripts; team members will review the coding and modifications will be made to the coding framework. Codes will then be organized into categories or themes to explain the data.

### Monitoring

At each peer coaching session, the peers will ask the participants if they have experienced any adverse events (e.g., falls, pain), and report it in the standardized form and communicate it to the research team. If a research coordinator notices any issues during contact with participants, they will refer the participants to their healthcare provider (e.g., family physician, physiatrist, prosthetist).

### Funding, ethics and dissemination strategy

This study has been funded by the Canadian Institutes of Health Research (CIHR). Research Ethics approval has been provided by the University Health Network. Any protocol amendments (e.g., changes to inclusion criteria, outcomes, or analyses) will be submitted to the ethics board as well as the clinical trial registry (NCT06667739 in Clinical Trial Registry (clinicaltrials.gov). De-identified data will be accessible upon a reasonable request after the findings are published. We have adopted an integrated knowledge translation approach. We will disseminate results through lay summaries, infographics, fact sheets, news stories, webinars, and relevant national and international meetings to reach multiple stakeholders. Tailored messages to target specific audiences will be produced.

## Discussion

The physiological, psychological, and social benefits of physical activity are well established [24]. Even minimal amounts of physical activity can improve functioning and slow the effects of deconditioning that are associated with disability [96]. Dysvascular LLA is characterized by high disability and low physical activity levels which may be a consequence of impairments from the amputation and comorbid conditions [27–30,42]. A multitude of barriers can restrict physical activity participation in this population including lack of knowledge, transportation, geographic barriers, co-morbid conditions, and availability of physical activity programs in the community [41,42]. To our knowledge, no studies have examined a peer-supported eHealth intervention to optimize physical activity in people with dysvascular LLA. This study will address this gap and pilot test a theory-informed intervention to optimize physical activity in people with dysvascular LLA prior to a larger study of intervention effectiveness. IMPACT-L3 has potential to deliver low-cost, accessible support for physical activity, ultimately improving the health and QoL of people with LLA.

## Supporting information

S1 File Interview Guide: Participants. (PDF)

S2 File Interview Guide: Peer Coaches. (PDF)

S1 Checklist Standard Protocol Items: Recommendations for Interventional Trials (SPIRIT) Checklist. (DOCX)
