# Supplementary material for: A behaviour change intervention promoting physical activity following dysvascular amputation: Protocol for a pilot study
Source: PLoS One. 2025 Jul 10;20(7):e0326761. doi: 10.1371/journal.pone.0326761 (PMC12244559; doi:10.1371/journal.pone.0326761)
Supplement: S1 File — (PDF) [file pone.0326761.s001.pdf]

JREB ID NUMBER: (office use only)

West Park Healthcare Centre-The Salvation Army Toronto Grace Health Centre
Joint Research Ethics Board
Research Application

Congruent with the Toronto Academic Health Sciences Council
Human Subjects Research Application

All sections of this application MUST be completed before it will be considered for REB review. If not applicable, indicate "N/A". Unless indicated, the Joint Research Ethics Board Application questions must be completed in the space provided. A complete application must be submitted to each site where this research will take place. A separate protocol must also be included with the application.

SECTION I: GENERAL INFORMATION

1. **SITE (where the study will be conducted):**

☒ West Park Healthcare Centre ☐ The Salvation Army Toronto Grace Health Centre

2. PRINCIPAL INVESTIGATOR NAME*:

Title: Dr	Last Name: MacKay	First Name: Crystal

3. FULL STUDY TITLE:

A Behaviour Change Intervention Promoting Physical Activity Following Dysvascular Amputation: A Pilot Study

4. SOURCE OF FUNDING:

Sponsor Name: CIHR
Sponsor Protocol Number (if applicable):
Granting Agency Name:
Internal Funding:
Other:

☒ Funding obtained ☐ Funding applied for (expected date of decision):

☐ **No funding required (explain):**

5. INVESTIGATORS:

A. PRINCIPAL INVESTIGATOR CONTACT INFORMATION

Dept/Div: Clinical Evaluation/Research Unit			Program:
Telephone: 4106-243-3600 x 4524	Pager:		Fax:
Street Address:<br>Line 1 82 Buttonwood Avenue			
Line 2			
City: Toronto	Province: ON	Postal Code: M6M 2J5	Email: Crystal.MacKay@westpark.org

PRINCIPAL INVESTIGATOR AGREEMENT - I assume full responsibility for the scientific and ethical conduct of the study as described in this REB application and submitted protocol and agree to conduct this study in compliance with the Tri-Council Policy Statement: Ethical Conduct for Research Involving Human Subjects and any other relevant regulations or guidelines. I certify that all researchers and other personnel involved in this project at this institution are appropriately qualified and experienced or will undergo appropriate training to fulfill their role in this project.

crystal MacKay ____________________ February 12, 2024 ____________________

Signature of Principal Investigator Date

B. CO-INVESTIGATOR(S):

Title	Last Name	First Name	Institution	Dept/Div/Program	Signature
Dr.	Miller	William	University of British Columbia	Occupational Science and Occupational Therapy	
Dr	Zidarov	Diana	Université de Montréal		
Dr.	Chan	Brian	Toronto Rehabilitation Institute-UHN		
Dr.	Dilkas	Steven	West Park Healthcare Centre	Division of Physical Medicine & Rehabilitation	
Dr.	Hitzig	Sander	Sunnybrook Research Institute		
Dr.	Marinho Buzelli	Andresa	Sunnybrook Research Institute		
Dr.	Mayo	Amanda	Sunnybrook Research Institute		
Dr.	Payne	Michael	University of Western Ontario		
Dr.	Schneeberg	Amy	Amy Schneeberg Statistical and Methods Consulting		

Dr.	Totosy de Zepetnek	Julia	University of Regina		
Dr.	Zucker-Levin	Audrey	University of Saskatchewan		

C. ON STAFF INVESTIGATOR (for studies initiated outside of the partnering institutions)*:
Not Applicable ☒

Title:	Last Name:		First Name:	
Dept/Div:			Program:	
Telephone:		Pager:		Fax:
Street Address:<br>Line 1				
Line 2				
City:	Province:		Postal Code:	Email:
Signature:			Date:	

***For studies initiated outside the four partnering institutions, the On Staff Investigator must be a West Park Healthcare Centre/The Salvation Army Toronto Grace Health Centre Staff Member or an external researcher with a formal affiliation/appointment with any of the partnering institutions.**

D. STUDY COORDINATOR OR RESEARCH ADMINISTRATIVE CONTACT FOR THIS APPLICATION (if not the PI):
Not Applicable ☒

Title:	Last Name:		First Name:	
Telephone:		Pager:		Fax:
Street Address:<br>Line 1				
Line 2				
City:	Province:		Postal Code:	Email:

Indicate to whom correspondence should be mailed: ☒ PI ☐ Administrative Contact

6. <u>FACULTY SUPERVISOR*</u> (for student/fellow/resident research studies):
Not Applicable ☒

Title:	Last Name:		First Name:	
Dept/Div:			Program:	
Telephone:		Pager:		Fax:
Street Address:<br>Line 1				
Line 2				

City:	Province:	Postal Code:	Email:
Signature:		Date:	

7. DIVISION/DEPARTMENT/PROGRAM APPROVAL

I am aware of this proposal and support its submission for ethics review. I consider it to be feasible and appropriate. I attest that the principal investigator responsible for this study has the qualifications and expertise to carry out this study in a competent and professional manner.

Jan Walker	VP Strategy, Innovation & CIO (Research)	JWalker	Feb 13/2024
Name (Print)	Div./Dept./Program (Print)	Signature	Date

8. STUDY PERIOD:

Expected Start Date: 02/2023 Total Study Duration: 2 years

9. INVESTIGATOR CLASSIFICATION

Staff Research: ☒ YES ☐ NO

Student Research: ☐ Post-Doctoral ☐ PhD ☐ Master's ☐ Undergraduate ☐ Resident/Fellow

Note: Where an investigator is a student/trainee, it is expected that the supervisor will be the Principal Investigator. If the supervisor is not on staff at the research site, check with your institution regarding who may be the PI.

Other (specify):

10. PRIOR ETHICS/SCIENTIFIC/SCHOLARLY REVIEW

Application submitted to (check all that apply):		Ethics Review and Approval Status (check all that apply and indicate date where applicable):			
		**Application To Be Submitted**	**Applied, Review Pending**	**Reviewed**	**Approved**
☐	West Park Healthcare Centre or The Salvation Army Toronto Grace Health Centre	☒	☐	☐	☐
	Other Institutions in the Toronto Area				
☐	Baycrest Centre for Geriatric Care	☐	☐	☐	☐
☐	Bloorview MacMillan Children's Centre	☐	☐	☐	☐
☐	Centre for Addiction and Mental Health	☐	☐	☐	☐
☐	Hospital for Sick Children	☐	☐	☐	☐
☐	Sinai Health	☐	☐	☐	☐
☐	Sunnybrook Health Sciences Centre	☐	☐	☐	☐
☐	Unity Health	☐	☐	☐	☐

☐	University Health Network	☐	☐	☐	☐
☐	Women's College Hospital	☐	☐	☐	☐
☐	Other (Specify)	☐	☐	☐	☐

***Include all relevant correspondence related to ethics review (i.e., REB review letter, replies, approval letter). If applying to more than one site, indicate which will be the primary site for ethics review:**

A. Has this proposal received prior scientific peer review? ☒ YES ☐ NO

If YES, indicate where and attach any relevant reviewer comments.
Scientific review from CIHR grants review
If NO, refer to institutional instruction page regarding possible review requirements.

B. Is this protocol associated (e.g. extension, roll over) with a previously approved study at this institution? ☐ YES ☒ NO
If YES, indicate:
Name of Principal Investigator:
REB file number:

11. MATERIAL TRANSFER AGREEMENT

Is there a material transfer agreement (MTA) involving human material for this study? (*This refers to an agreement for transfer of biological materials (e.g., tissues, cell lines) from the institution to another institution or other entity.*) ☐ YES ☒ NO
If YES, attach a copy.

12. INVESTIGATIONAL DRUGS OR DEVICES

Not Applicable ☒

A. Does this study involve any of the following (check all that apply):
- ☐ Investigational New Drugs
- ☐ Investigational Biologics
- ☐ Investigational Natural Health Products (NHP)
- ☐ Investigational Medical Devices
- ☐ Approved drug for a new indication (e.g., new age-group, disease entity)?

B. If the study involves any of the above:
Is "No objection" or authorization letter from Health Canada attached? ☐ YES ☐ NO
If no, has a Clinical Trial Application (CTA) been submitted (or will soon be submitted) to Health Canada? ☒ YES ☐ NO

If pending, provide date of submission:

Health Canada "No Objection" file #:

If "No objection" letter or authorization is pending, forward approval letter to the REB office as soon as it is available.

C. Provide FDA IND number (drug studies) or PMA number (device studies):

☒ Not Applicable
☐ Pending (if pending, forward to the REB office when available)

SECTION II: STUDY SUMMARY

NOTE: THIS IS NOT A SUBSTITUTE FOR THE FULL PROPOSAL.

13. <u>ABSTRACT</u>

Must be a summary of study **suitable for lay audience**.

(Max. 100 words.)

Loss of a lower limb due to diabetes can negatively impact physical and mental health and quality of life. Individuals are at risk of other diseases such as cardiovascular disease, loss of the other limb and death. Physical activity can improve health outcomes, but activity levels are low in this group. Rehabilitation services are not readily available and costs of programs are high. We worked with this community to create a virtual, peer-led physical activity behaviour change intervention called IMproving Physical Activity through Coaching and Technology following Lower Limb Loss (IMPACT-L3). We will conduct a pilot study to assess feasibility and optimize program design to be studied in a larger future trial.

14. <u>RATIONALE AND HYPOTHESIS/RESEARCH QUESTION</u>

Include the significance of the study.

(Max 1/2 page)

Diabetes-related lower limb amputation (LLA) is a leading cause of disability globally.[1] In the US alone, 185,000 LLAs are performed annually and the number of LLAs are projected to double by 2050 as a result of increasing rates of diabetes and the aging population.[2] In Canada, 7,300 people have a LLA each year.[3] More than 80% of these amputations result from complications of diabetes and/or peripheral vascular diseases (dysvascular LLAs).[3] While dysvascular LLA commonly affects adults over age 65, there has been a resurgence of diabetes-related amputations in younger and middle-aged adults.[4] LLA is a significant life event which impacts mobility, mental health and ultimately quality of life.[5-10] In addition to disability resulting from LLA, individuals with dysvascular LLA often have multimorbidity (mean of five health conditions)[11 12] and 37% of individuals require a contralateral or revision amputation within 5 years which can further impact their function and quality of life.[13] Moreover, this population has a poor survival rate, estimated between 17 months and 4 years [14-18] with cardiovascular comorbidities cited as the leading cause of death.[18-25] Due to their complex health challenges, people with dysvascular LLA have high rates of health care utilization (50% readmission rate)[26] leading to high healthcare costs. While Canadian cost estimates are unavailable, healthcare costs for amputation in the US are >4.3 billion annually.[27] With such high costs, rehabilitation services for people with LLA are often limited.[28 29] In Canada, only 18% of adults with LLA receive inpatient rehabilitation and services are more available in urban than remote areas.[30] These findings underscore the need for accessible, low-cost approaches to optimize health, social participation and quality of life in this population.

There is strong evidence that adequate physical activity reduces risk of chronic disease, all-cause and cardiovascular mortality and improves quality of life.[31] Moreover, physical activity is a cornerstone of management of LLA[32] and may confer additional benefits for people with dysvascular LLA such as improving diabetes outcomes and prevention and management of cardiovascular disease.[31 33] Unfortunately, dysvascular LLA is characterized by high disability and low physical activity levels which may be a consequence of the amputation and comorbid conditions.[36 39 41] People with dysvascular LLA have reduced strength, balance, cardiorespiratory fitness, walking ability and increased energy expenditure during ambulation.[35 38 53 54] Consequently, they have impaired mobility leading to lower physical activity engagement. The presence of comorbidities and secondary conditions including skin problems (e.g., pressure ulcers)[55-58] and musculoskeletal conditions (e.g., low back pain) can further impact individuals physical activity levels.[59] Across studies examining physical activity in people with dysvascular LLA, step counts ranged from 1250 steps/day in older prosthesis users to 3809±2189 steps/day in people with diabetes-related LLA.[40 60-62] People with dysvascular LLA accumulated 24±41 minutes per week of moderate to vigorous physical activity, the minimum recommended 150 minutes per week.[40] Importantly, not everyone with LLA can use a prosthesis due to muscle weakness, cognitive impairment or skin problems.[63 64] It can be difficult for wheelchair users to accrue adequate physical activity.[65]

Given the limited availability of rehabilitation services for LLA, accessible interventions to support physical activity in people with LLA are needed. Evidence suggests that multifaceted interventions that incorporate social support and behaviour change techniques increase physical activity in older adults and people with chronic illness.[34-37] In a national meeting, stakeholders ranked research on peer led physical activity programs for people with dysvascular LLA as a top research priority.[38] To date, no peer-led physical activity interventions for people with dysvascular LLA have been evaluated.[39 40]

To address this gap, we co-created a novel physical activity behaviour change intervention comprised of peer coaching, wearable technology and education. Prior to conducting a definitive trial to determine effectiveness of the intervention, a pilot study is required to assess feasibility and optimize design of a future trial.

The **primary aim** is to assess the feasibility of conducting a definitive randomized controlled trial to determine the effectiveness of a virtual peer-led physical activity intervention on levels of physical activity and self-efficacy compared to a wait-list control in people with dysvascular LLA.

Specific objectives are to:

1) Evaluate feasibility according to indicators of process, resources, management and treatment.
2) Explore perceptions of program characteristics, program implementation and study procedures among individuals with LLA.
3) Explore the perceived feasibility and acceptability of the program among peer coaches.
4) Inform a sample size calculation for a future trial of effectiveness.
5) Assess construct validity of physical activity and self-efficacy measures among individuals with dysvascular LLA.

15. STUDY DESIGN

(Many of these questions apply to clinical research studies. If any of the items are not applicable to your study, indicate N/A):

A. Describe Design/Methodology.

Indicate Clinical Trial Phase (I, II, III, IV) where appropriate.

(Max 1 page)

Design: This pilot study is a parallel group RCT with an embedded qualitative component. A pilot study is a subset of feasibility studies which asks questions about feasibility (whether the future trial can be done and, if so, how) but with a key design feature: in the pilot study, the future RCT is conducted on a smaller scale. Prospective recruitment, concealed group allocation, evaluator masked outcome evaluation and waitlist control will be employed. The research is guided by the MRC framework for evaluating complex interventions.[51] We will follow the extension to the Consolidated Standards of Reporting Trials Statement for reporting pilot and feasibility RCTs.[87]

Participants: Individuals who meet the following inclusion criteria will be included: 1) Dysvascular LLA (LLA due to diabetes or vascular disease); 2) major LLA (at the ankle or above); 3) living in the community; 4) adult at least 18 years of age; 5) comfortable communicating in English and able to understand basic English; and 6) receptive to using a phone or tablet (e.g., to enable peer coaching, access to modules. Participants may be ambulatory with a prosthesis and/or use a wheelchair.

Exclusion criteria include: 1) actively receiving rehabilitation services related to physical activity/mobility; 2) recommended medical supervision for physical activity by health care provider, [102] or skin problems preventing usual activity and 3) not able to provide informed consent.

Participants who are interested in taking part in the study but who do not have access to a device will be offered the use of a device to borrow. The number of participants who may be offered the use of a device may be limited. Participants who may require additional support with the use of technology will be provided such supports to increase accessibility. This support may include instructions and support with accessing and signing in to Zoom/WebEx and walking through the use of Zoom/WebEx to facilitate peer coaching sessions and/or accessing and walking through the online modules and/or study measures.

RCT Group Allocation: Participants will be assigned to one of two groups: intervention group (see details below) and control group (wait list control). Randomization stratified by age and sex will be employed with permuted block randomization of varying block size (2 and 4) to reduce the possibility of selection bias. The allocation schedule will be created using an online tool by a person outside of the research team who is not involved in recruitment. Age is a predictor of physical activity[85] and could be associated with the efficacy of the intervention. The age groupings (>65, 65+) are consistent with research demonstrating differences in physical activity by age in people with LLA.[101] Stratification will be employed to ensure representation across age ranges and sex to enable investigation of these variables as effect modifiers.

An independent statistician will provide the randomization list through REDCap (REDCap Software, Vanderbilt University and National Institute of Health, USA). Upon enrolment, a study ID number will be allocated to each participant. The study ID number will be linked to the randomization list.

Allocation concealment: After completing the baseline assessment, the research coordinator

will reveal the group allocation of the participant through the REDCap randomization list.

Masking: Due to the nature of the intervention, masking to receipt (or not) of the intervention is impossible. In this study, one assessor will collect all data. They will be masked to group allocation. We will ask participants not to disclose their group allocation during assessments.

Qualitative Interviews: For objectives 2 and 3, we will use a qualitative descriptive approach [79] to understand participants' and peers' experiences with IMPACT-L3. The research will be situated within an interpretive research paradigm.[80] This component will be critical to improving the intervention and refining the protocol for the definitive trial. To explore perceptions of recruitment approaches, data collection procedures and measures, time burden, and acceptability of program implementation (Objective 2), one-on-one semi-structured telephone or Zoom interviews (Interview guides for peer health coaches and people with limb loss: Appendix G) lasting ~45-60 minutes will be conducted with participants after completion of the intervention. Participants will be asked to share their perceptions of program characteristics. For example, participants will be asked about their perceptions of Apple Watch as a tool to support behaviour change including the appropriateness of the data provided (e.g., distance) and the usability of the device. Findings will inform refinements of the intervention, if needed (e.g., development of a custom app to use with Apple Watch). Data will be collected until data saturation. Based on prior research[81], we anticipate recruiting ~15-20 participants. We will sample purposively for variation in age, gender and level of amputation. For objective 3, we will conduct semi-structured interviews with all consenting peers to understand their experiences and identify considerations for optimizing the intervention.

B. What are the primary outcome measures?

☐ Not applicable

The primary outcome of this study is feasibility of implementing the intervention and conducting the trial.

Feasibility Measures (Appendix D):

Process Indicators: *Recruitment rate* will be evaluated by the number and proportion of participants recruited per month and the number and proportion of peers recruited. *Consent rate* will be calculated as the percent of recruited individuals who provide consent per month. *Withdrawal rate* will be calculated as the percent of study participants withdrawing by post intervention at 9 weeks (T2) and 4 weeks later (T3). *Acceptability* will be assessed in qualitative interviews and by the theoretical framework of acceptability (TFA),[103] a brief questionnaire developed to assess acceptability in the design, evaluation and implementation of interventions.

Resource Indicators: *Participant adherence* will be measured as the percentage of peer coaching sessions participants attend. Participants' usage of web-based modules will be measured (i.e., number of completed modules, number of logins). *Peer coach adherence* will be assessed by tracking the total number of peer coaching sessions attended by the peer-trainer. *Participant and tester burden* will be measured by the amount of time it took to administer study outcomes at T1, T2, and T3 and the acceptability of the evaluation time commitment from the perspective of participants.

Feasibility of data collection will be evaluated as the percentage of participants with complete data

on each measure at each evaluation time point. For accelerometers, the percentage of devices that were returned at baseline and follow-up (9 weeks, 3 months) and the amount of valid wear time will be assessed.

Management Indicators: *Participant processing time* will be assessed as time from initial contact to enrolment. *Intervention fidelity* will focus on adherent and competent delivery of the intervention. It will be evaluated using the study-specific checklist outlining key components of the intervention completed by peers. A subset of peer coaching sessions (at least one per dyad) will be recorded and reviewed by research staff using the checklist.

Treatment Indicators: *Adverse events* will be measured as the number of adverse events that occurred during physical activity for the intervention. Adverse events (e.g., falls) will be documented by peers on the standardized form used at each coaching session.

Proposed Primary outcomes for the main trial (Appendix D)

While this pilot trial is not powered to detect meaningful differences in these outcomes, collecting data on our proposed outcomes can assess and ensure that there are no issues with the collection and completion of these measures in preparation for a future trial.

Objective Physical Activity (Accelerometer). A tri-axial accelerometer is a lightweight device used to measure total activity counts. The total volume of physical activity (activity counts) will be measured. This measure has the advantage of integrating the frequency, intensity, and duration of movement and combining them into an overall measure of physical activity.[61] The sum of the total count for the day will be used and averaged over the measurement period. Total activity counts can be a better metric than the number of minutes per day spent in various physical activity intensity categories because it incorporates all levels of intensity. Recent evidence suggests that light, moderate and vigorous activity **all** have health benefits.[62] [63] Ambulatory participants will wear the monitor on a waist belt on the side of the shortest residual limb which provides the most valid data in people with LLA.[64] Wheelchair users will wear ActiGraph™ wGT3X-BT on the non-dominant arm.[65] For wheelchair users a second ActiGraph™ placed on the rear wheel is recommended (waterproof box installed on the rear wheel using tie wraps). Participants will wear the accelerometer at all times except while bathing or swimming for a period of 7 days pre- and post-intervention and 4 weeks later. Only data from days in which participants wear the activity monitors for >10 hours per day will be included in the analyses as per previous approaches, including individuals with dysvascular amputation.[42]

Self-efficacy for Exercise Scale. A self-report measure that includes situations that may influence physical activity participation. Participants will respond to each item on a 0 (not very confident) to 10 (very confident) scale. This is a valid measure of exercise self-efficacy in older adults.[66]

See full protocol for **secondary outcome measures**.

C. Is a placebo used in this study? ☐YES ☒ NO

If YES, how is this justified (e.g., no alternative standard treatment available)? Include any provisions in place to reduce risks to subjects assigned to placebo (e.g., increased monitoring, rescue medication). (Max ¼ page)

D. Does the study involve deception or intentional lack of disclosure? ☐ YES ☒ NO

If YES, explain justification and how subjects will be debriefed.

(Max ¼ page)

E. Will the subject be withdrawn from or denied usual therapy for any condition in order to participate in the study or be subject to other restrictions? ☐ YES ☒ NO

If YES, explain.

(Max ¼ page)

16. **SUBJECTS/CONTROLS**

A. How will subjects be chosen (main inclusion/exclusion criteria)?

If applicable, how was the proposed control group selected?
(Max ¼ page)

Potential participants will self-identify or be identified by a member of the research team or health professional. Individuals with dysvascular LLA will be recruited from rehabilitation hospitals with support from collaborators. Patients who have completed rehabilitation services as an inpatient or outpatient following LLA often continue to have long term follow-up appointments with the clinical team (e.g., annual visit, assessment of prosthesis, etc). Participants will be recruited from rehab clinics in the community, for example, prosthetics clinics, as well as primary healthcare clinics. Potential participants will also be recruited through community centers (e.g., Variety Village) and the networks of the research team. A research database of individuals who have indicated in past research their interest to take part in future studies will be used to identify potential participants. Individuals who have consented to being contacted for future study participation may receive a Study Invitation Letter.

Study posters will be used to recruit potential participants. Study posters will be displayed in the clinical practice areas and in outpatient amputation clinics at West Park Healthcare Centre, at rehabilitation centres/hospitals, at other clinic sites that treat individuals with a LLA (e.g., St John's Rehab), patient organizations, community programs and organizations (e.g., Variety Village), on amputation and/or limb loss websites (i.e., Amputee Coalition of Canada), social media accounts of partners including but not limited to Amputee Coalition of Canada, newsletters, listserv/contact listservs of affiliated organizations and networks of the research team across Canada.

Social media advertisements will be used to recruit potential participants. Social media ads will be displayed on West Park social media and websites (e.g. Twitter, Facebook, WP website) and other hospital, health centre, or affiliated organizations' social media accounts and websites.

Study information sheets and study invitation letters will be used to recruit potential participants. Clinicians at the rehabilitation hospitals will be informed about the study via a Study Information Sheet explaining the project. Study Invitation Letter will be provided to potential participants.

Inclusion Criteria: 1) Dysvascular LLA (LLA due to diabetes or vascular disease); 2) major LLA (at the ankle or above); 3) living in the community; 4) adult at least 18 years of age; 5) comfortable communicating in English and **a**ble to understand basic English; and 6) receptive to using a phone or tablet (e.g., to enable peer coaching, access to modules.. Participants may be ambulatory with a prosthesis and/or use a wheelchair.

Exclusion criteria include: 1) actively receiving rehabilitation services related to physical

activity/mobility; 2) recommended medical supervision for physical activity, by healthcare provider or skin problems preventing usual activity and 3) not able to provide informed consent.

i. What is the age range of eligible subjects?
Must be at least 18 years of age

B. Number to be enrolled at this institution: 60
Total study enrolment: N = 60 (30 per arm)

C. Approximate size of eligible population from institution/practice:
Approximately 200 patients with amputations attending inpatient rehabilitation at West Park Healthcare Centre annually. Approximately 20 patients per week attend outpatient follow-up in the ambulatory care clinic.

D. Is sample size justified in the protocol? ☒ YES ☐ NO
If NO, provide sample size justification.
(Max ¼ page)

17. STUDY INTERVENTIONS or PROCEDURES INVOLVING HUMAN SUBJECTS

Not Applicable (e.g. observational studies). ☐
If not applicable, go directly to 16. DATA ANALYSIS.

A. Usual standard of care.
Document what is the usual standard of care at this institution for this population.
Not Applicable ☐

(Max ½ page)

Following amputation, patients may attend inpatient and/or outpatient rehabilitation at West Park Healthcare Centre. Following rehabilitation, patients may have regular follow-ups for assessment of the prosthesis.

B. Changes/additions to usual standard of care.
Indicate what procedures are to be carried out in the study, that are NOT considered part of the diagnostic, therapeutic "routine" or standard care of the subject or how standard care is altered. Attach a copy of all instruments (i.e., questionnaires, rating scales, etc.)

(Max ½ page)

The intervention and data collection for the study will be virtual (not be conducted at West Park Healthcare Centre) and therefore participation in the study will not affect any routine standard care or require extra commitment or other procedures as part of their care at West Park Healthcare Centre.

Experimental Intervention (IMPACT-L3): Theory-based interventions are more effective in increasing physical activity.[48] Two theories provide a framework for IMPACT-L3. One is social cognitive theory, which is a useful theoretical lens for incorporating self-efficacy into interventions.[49 50] Self-efficacy is the belief a person has in his or her ability to perform a behavior successfully.[51] Self-efficacy is informed by skill mastery, vicarious experience, verbal persuasion, and reinterpretation of physiological and affective symptoms.[51] The other is self-determination theory, which provides a framework for cultivating an autonomy-supportive social environment that promotes behavior change.[52] This is achieved by satisfying three basic psychological needs of autonomy (i.e., volition in one's own behavior), competence (i.e., interacting effectively with one's environment by mastering tasks), and relatedness (i.e., sense of belonging).[52] These constructs were integrated into IMPACT-L3. As eHealth solutions have the potential to increase access and improve health outcomes,[53 54] IMPACT-L3 will be delivered entirely virtually. See Appendix A for logic model.

Peer health coaching (~30 minutes weekly) will be delivered by a peer trainer over 8 weeks. Peer trainers, who have experienced a dysvascular LLA themselves, will be matched to a participant based on gender and level of amputation to promote a sense of understanding and belonging. Individuals with higher level amputations (i.e., above knee) are less physically active and face more challenges with mobility (e.g., greater energy expenditure)[55 56] and may benefit from a peer with similar experience. Peers will be trained to implement the intervention including specialized training in brief action planning (BAP), a structured support technique grounded in the principals and practices of motivational interviewing (Appendix C). Training will be delivered by an organization which provides certified training in BAP (Centre for Collaboration, Motivation and Innovation)[57] including an online course and practice and feedback. The trained peer will help participants **set goals and create an action plan for physical activity** that they feel confident that they can achieve. During weekly meetings, peers will help participants problem solve challenges to physical activity and overcome barriers. The peer trainer will deliver the intervention through **voice or video calls** (depending on preference). Video calls will be preferred because "face-to-face" interactions may reinforce vicarious experiences (e.g., peers demonstrate movements). Peers will complete a standardized form at every interaction to review goals, goal progression (e.g., Apple Watch data), action plans, and document barriers/facilitators. Peer health coaches will be asked to complete a standard form for each coaching session (Appendix D). Weekly debriefings among peers and the research team will problem solve challenges and monitor peer burden.

To improve competence and skills to enhance physical activity, participants will have access to five web-based modules developed based on qualitative interviews and co-design workshops with people with LLA. Modules include content on physical activity including benefits of physical activity, types of physical activity (strengthening, aerobic, balance, flexibility), intensity of activity (light, moderate, vigorous), exercise safety and limb management to enable physical activity, and recommended physical activity. The written materials that correspond with the online modules is included in Appendix E. The modules

will be housed on a learning management system at the University of Toronto. They will include videos. Participants will be given a login and will be asked to review modules at their own pace each week for the first 5 weeks.

To support behaviour change, participants will be provided with a wearable to track their physical activity: an Apple Watch to be worn at the wrist of the non-dominant side. An off the shelf wearable was selected for the study **to optimize sustainability of the intervention in a real-world setting**. Apple Watch has been shown to be accurate in tracking step count in a range of populations[58] and can measure wheelchair pushing thus making it the best option available.[59] Participants will be trained to wear and use the Apple Watch 24 hours a day during the intervention time only, including water-based activity. Wearables that provide personalized, and actionable feedback promote better behavior change outcomes.[60] Data from the Apple Watch will be shared verbally with the peer during discussions between participants and peers during the weekly virtual sessions to facilitate behaviour change.

Control: The control group will continue with their usual health care and be offered the intervention program at the end of the follow-up period (wait-list control).

C. What are the additional risks associated with the study as compared to usual standard of care?
Do not refer to other sections of this form.
(Max ½ page)

There are minimal additional risks to participants in the study, which are inherent to physical activity participation. However, the long-term benefits outweigh those risks. Telephone-based physical activity interventions have been shown to be safe for people with dysvascular LLA in RCTs. To enhance safety with physical activity (e.g. reduce risk of falls, skin problems), LLA-specific education modules will address exercise safety and peers will be trained in how to address risks.

Some participants may feel uncomfortable answering interview questions about their health. All participants will be informed they are not required to respond and may "pass" any questions they do not wish to address.

D. Subject Time Commitments.
Indicate duration of study visits or extra time commitment (length, number, and frequency of test sessions) for study participation.

Participants will not require any additional time at West Park Healthcare Centre as part of their usual care.
Participants will take part in 30 mins of peer health coaching weekly for 8 weeks. For the first 5 weeks, participants will also complete modules for approximately 30 minutes. All participants will complete 3 assessments including an online questionnaire estimated to take approximately 30-45 minutes to complete. Some participants will be asked to complete a one-on-one interview lasting approximately 45-60 minutes.

18. DATA ANALYSIS

Briefly explain what methods will be used to analyse study data.
You may refer to protocol for this question.

See below.
(Max ¼ page)
Data Analysis plans are described in the protocol in section 3.11 on Page 12.

SECTION III: ETHICAL ISSUES

19. RECRUITMENT AND CONSENT

Note: Any document to be viewed by the subject (e.g. consent/assent forms, information sheets, recruitment posters/letters) must be included with your submission. Refer to the other materials in this package for more detailed instructions.

A. How will potential subjects be identified and/or referred?

☒ Healthcare professional
☐ Permanent Health Record/Clinical Chart
☒ Other Existing Database (specify): Clinic database at West Park Healthcare Centre

☒ Advertisements, including web based recruitment tools (attach a copy if applicable)
☐ Other (specify):

i. Indicate who will identify potential subjects.
(Max ¼ page)

Potential participants will self-identify or be identified by a member of the research team or health professional. Individuals with dysvascular LLA will be recruited from rehabilitation hospitals with support from collaborators. Patients who have completed rehabilitation services as an inpatient or outpatient following LLA often continue to have long term follow-up appointments with the clinical team. Participants will be recruited from rehab clinics in the community, for example, prosthetics clinics, as well as primary healthcare clinics. Potential participants will also be recruited through community centers (e.g., Variety Village) and the networks of the research team. A research database of individuals who have indicated in past research their interest to take part in future studies will be used to identify potential participants. Individuals who have consented to being contacted for future study participation may receive a Study Invitation Letter.
Study posters will be used to recruit potential participants. Study posters will be displayed in the clinical practice areas and in outpatient amputation clinics at West Park Healthcare Centre, at rehabilitation centres/hospitals, at other clinic sites that treat individuals with a LLA (e.g., St John's Rehab), patient organizations, community programs and organizations (e.g., Variety Village), on amputation and/or limb loss websites (i.e., Amputee Coalition of Canada), social media accounts of partners including but not limited to Amputee Coalition of Canada, newsletters, listserv/contact listservs of affiliated organizations and networks of the research team across Canada.

Social media advertisements will be used to recruit potential participants. Social media ads will be displayed on West Park social media and websites (e.g. Twitter, Facebook, WP website) and other hospital, health centre, or affiliated organizations' social media accounts and websites.

Study information sheets and study invitation letters will be used to recruit potential

participants. Clinicians at the rehabilitation hospitals will be informed about the study via a Study Information Sheet explaining the project. Study Invitation Letter will be provided to potential participants.

See Appendix F for all recruitment materials.

ii. Explain how enrollment in multiple studies is managed in this patient population at this institution.
Not Applicable ☐
(Max ¼ page)

Potential participants will be asked if they are participating in any other studies. If so, they will be asked if they still want to participate. If they do not indicate undue burden, they will be allowed to enroll in the study.

B. Explain who will make initial contact with subjects or authorized third party and how (e.g. in person, phone, letter, e-mail/web site). Attach a copy of the script or any written materials if applicable.
(Max ¼ page)

A research coordinator will have initial contact with potential participants. Individuals who self-identify and contact the study team via phone or email will receive a response from the research coordinator who will review the study information and determine their study eligibility. Potential participants may have initial contact with a health professional within their circle of care or a research coordinator or administrator during an in person or virtual clinic visit. They may receive a Study Invitation Letter and may be asked by clinical or administrative staff if they agree to being contacted by a research coordinator to discuss the study. All appendices attached.

C. Describe the consent process. (E.g., Will consent be written, oral, telephone (include script), and who will obtain consent.) *If the study population requires special consent considerations (e.g. child, incompetent adult, unable to communicate) you may refer to item E. of this section.*

(Max ¼ page)

Individuals who agree to participate and are eligible will have the opportunity to ask questions before reviewing and signing the Consent Form. Participants will sign a Consent Form prior to taking part. Consent Forms will be signed and returned online via REDcap. Individuals who are not able to sign the Consent Form via REDCap will be given an option to sign and return the Consent Form in person, via email or by mail with a prestamped and addressed envelope. Potential participants will be given as much time as they need to decide if they would like to participate, up until the time that the study activities are scheduled to occur.

Upon obtaining consent, the research coordinator will schedule the first online meeting with a blinded assessor to complete the baseline assessment. The research coordinator will log onto the online randomization system to determine the next allocation. The research coordinator will forward the participant's contact information to schedule the first online meeting.

i. How much time will be given to subjects to review the information before being asked to give consent?

Potential participants will be given as much time as they need to decide if they would like to participate, up until the time that the study activities are scheduled to occur.

D. Is there a relationship between the subjects and:

Person obtaining consent ☐ YES ☒ NO

Investigator ☐ YES ☒ NO

If YES, explain the nature of the relationship (e.g., physician, employer) and what steps will be taken to minimize a potential perception of coercion.

E. Will this research involve any of the following? (Check any that apply):

☐ genetic research
☐ tissue samples
☐ healthy volunteers
☐ students
☐ staff
☐ prisoners
☐ involuntary subjects
☐ emergency patients
☐ women of child-bearing potential
☐ pregnant women
☐ children less than 16 years of age
☐ fetal tissue or placenta
☐ incompetent subjects
☐ borderline incompetent subjects
☐ subjects unable to communicate
☐ individuals who may require translation or who are illiterate
☐ individuals temporarily unable to provide an informed consent (e.g. unconscious, emergency?)

☒ none of the above

The above list identifies research that may require special consideration, e.g. regarding confidentiality, voluntariness, risk or capacity to consent. If the research will involve any of the above attach a summary explaining how the subject's interests will be protected, how capacity will be determined (if applicable) and how surrogate consent and assent (if applicable) will be obtained. Where inability to provide an informed consent is expected to be temporary, describe what plans are in place to regularly assess capacity and to obtain consent if the individual later becomes capable of providing consent. For subjects who have limited skills in English or are illiterate, attach a summary explaining what special procedures are in place (e.g.,

translated forms, translator, impartial witness).

20. RISK/BENEFIT ESTIMATES

A. Potential Benefits to Subjects

List anticipated benefits if any. ☐ No direct benefits anticipated.

Participants may or may not benefit from participating in this study. The intervention provided through the online education modules, peer coaching and an activity tracker may benefit some participants and help them to improve physical activity levels.

B. Potential Harms (Injury, Discomforts and Inconveniences) to Subjects (including psychological factors):

i. Document the risks to subjects involved in this research. ☐ NO known risks
(Max ¾ page)

There are no anticipated major risks beyond what is expected in normal life when participating in the study. Telephone-based physical activity interventions have been shown to be safe for people with dysvascular LLA in RCTs. To enhance safety with physical activity (e.g. reduce risk of falls, skin problems), LLA-specific education modules will address exercise safety and peers will be trained in how to address risks.

During interviews, some participants may feel uncomfortable answering questions about their health. All participants will be informed they are not required to respond and may "pass" any questions they do not wish to address.

While the risk is low, participants could experience distress, for example, while completing assessments. If any participants exhibit signs of distress while completing the assessments, the research team will follow up with those participants and if needed, provide them with a list of mental health resources (Appendix I).

a. For studies involving placebo, washout, or withholding of treatment, indicate risks related to absence of treatment. Not Applicable ☒

b. Include a summary of the data regarding reproductive risks such as teratogenicity or embryotoxicity of the study drug, any risk with breastfeeding, or risk to men regarding conception.
Not Applicable ☒
(Max ¼ page)

ii. Does participation in this study affect alternatives for future care? (e.g., development of antibodies that could prohibit future treatment with this or similar compounds) ☐ YES ☒ NO
If YES, explain.
(Max ¼ page)

21. PAYMENTS TO SUBJECTS

Indicate what payments, if any, will be provided to subjects:

☐ Reimbursement for expenses incurred as a result of research. Amount: $
Specify (e.g., travel, meals)

☒ Gifts for participation Participants will receive a gift certificate valued at $50 for each of the 3 assessments. Twenty participants and/or peer coaches will be asked to take part in a qualitative interview. All those who complete an interview will receive a gift certificate valued at $50. Participants will be able to keep the AppleWatch at the end of the study.

☐ Compensation for time Amount: $
If compensation for time will be provided, please justify:

22. MONITORING

A. Is there a steering committee? ☒ YES ☐ NO ☐ Not Applicable

B. Is there a plan for monitoring of the study (e.g., sponsor-initiated site visits)? ☐ YES ☐ NO ☒ Not Applicable
If YES, describe:
(Max ¼ page)
A Data and Safety Monitoring Board (DSMB) will review accumulating outcome data and advise the investigators regarding safety issues, evidence of benefit, and need for modification to the study design. The DSMB will include at least 3 members external to the research team: a statistician, a clinician and an individual with LLA. Adverse events will be documented.

C. Is an interim analysis planned? ☐ YES ☒ NO
If YES, describe briefly.

D. Is there a data and safety monitoring board (DSMB). ☒ YES ☐ NO
If NO, please justify:

If YES, is it independent of the sponsor? ☒ YES ☐ NO

23. POTENTIAL CONFLICTS OF INTEREST
Does the principal investigator or any co-investigators involved in this research study or any member of their immediate family:

☐ Function as an advisor, employee, officer, director or consultant for the study sponsor?

☐ Have direct or indirect financial interest in the drug, device or technology employed (including patents or stocks) in this research study?

☐ Receive an honorarium or other personal benefits from the sponsor (apart from fees for service)?

☒ **None of the Above**

If any of the above conflicts apply, append a letter to the Chair of the REB, detailing these activities and how

they will be managed. Disclose all contracts and any conflicts of interest (actual, apparent, perceived, or potential) relating to this project.

24. PUBLICATION /DISSEMINATION OF RESULTS

A. Is there an independent steering committee regarding publication? ☐ YES ☒ NO

B. How will the results be communicated to subjects and other stakeholders (e.g. advocacy groups, scientific community)?
Check all that apply:

☐ Individual debriefing at end of test session ☒ Publication (e.g., journal article, presentation)

☐ Group debriefing ☐ No plan

☐ Letter of appreciation at end of study

☒ Other (specify): Lay summaries for participants, meetings with partners and collaborators, newsletters, news stories, webinars, briefing reports, lay summaries, infographics, fact sheets

SECTION IV: FUNDING and CONTRACTS

25. BUDGET

Attach an itemized study budget (applies to full board and expedited review studies).

Do the funds presently available or applied for cover all requirements to conduct the project? ☒ YES ☐ NO

If NO, explain how the shortfall will be made up:

26. CONTRACT/RESEARCH AGREEMENT

☒ **No Contract/Research Agreement Involved**

☐ **Contract/Research Agreement Involved**
Name of sponsor/agency:

Has the contract/research agreement been submitted for review and signing (see institution specific instruction page)? ☐ **YES** ☐ **NO**

A. Liability

i. Is there external (non-institutional) liability insurance? ☐ YES ☒ NO

ii. If the subject suffers an injury as a result of participation in the study, who will cover reasonable out-of pocket expenses to ensure that immediate medical care is provided?
☐ Sponsor ☒ Institution
☐ Other (specify):

B. Publication Agreements

i. Is there an agreement between the investigator and the sponsor regarding use, publication or disposal of the data? ☐ YES ☒ NO

If YES, does the funding agency or sponsoring company place any restrictions on publication of findings or reporting of interim results? ☐ YES ☐ NO

If YES, explain any restrictions.

ii. Does the contract/research agreement permit the disclosure of research results, including SAEs, to stakeholders (subject and/or guardian, sponsor, REB, REBs of other sites, and regulatory agencies) if required to protect the health of subjects? ☐ YES ☐ NO

SECTION V: PRIVACY AND CONFIDENTIALITY

27. PRIVACY AND CONFIDENTIALITY

Under the Personal Health Information Protection Act (Bill 31) which came into force in Ontario on Nov. 1, 2004, the following information must be provided to the Research Ethics Board (REB) when requesting approval of research studies involving the collection, use and disclosure of personal health information.

A. Describe all personal health information required to be collected and the potential sources of this information. If subject identifiers will be used on data collection forms (e.g., names, initials, DOB, OHIP #, Hospital ID# etc.), provide justification.
(Max 1/3 page)

Personal information (names and contact information) will only be used for administrative purposes (i.e., scheduling an interview). Demographic data will be used to describe the study population (e.g., mean age, number of male/female participants). Demographic data from individuals with amputations will include: date of birth (month and year), city of residence, country of birth, gender and sex, level of amputation, date and cause of amputation, date of admission and discharge, level of education, work status and living arrangements, comorbidities, primary mode of mobility, etc.

B. Describe how the personal health information will be used in the research.
(Max 1/3 page)

PHI will be used for administrative purposes and to describe the study population (i.e. mean age, number of male/female participants, etc).

C. If personal health information is to be linked to other information, provide the following details: **NA** ☒

i) Describe the information that the personal health information will be linked to.

ii) Explain how the linkages will be made.

iii) Explain why these linkages are required.

D. Explain why the research cannot reasonably be accomplished without using personal health information.
(Max 1/4 page.)

PHI is needed for administrative purposes to contact potential participants and schedule the interviews and to adequately describe the sample.

E. If consent to the disclosure of the personal health information is not being sought from the individuals to whom the information relates, provide justification as to why it would be impractical to obtain explicit consent.

No data without consent will be collected, used, or disclosed.

F. Describe the *reasonably foreseeable* harms and benefits that may arise from the use of the personal health information, and how the harms will be addressed.
(Max ¼ page)

No foreseeable harms and benefits may arise from the use of PHI. However, if personal information is inappropriately released it may cause the participants embarrassment. The REB will be notified immediately if personal information is inappropriately released.

G. Describe all persons who will have access to the personal health information, their roles in relation to the research and reason for access, and their related qualifications.

Name	Institution	Qualifications	Role/Reason for Access
Crystal MacKay	West Park Healthcare Centre	PhD	Conduct of Study
Parminder Flora	West Park Healthcare Centre	PhD	Conduct of Study
Angela Tobia	West Park Healthcare Centre	MSc	Conduct of Study

H. i) Describe the safeguards that will be imposed to protect the confidentiality and security of the personal health information.
(Max ¼ page)

Data will be password protected and stored on the West Park Healthcare Centre network drive that has firewalls and security measures in place. Hard copy records will be stored in a locked cabinet in a secure location at West Park Healthcare Centre. Access to records and data will be limited to authorized persons.

Study data will be de-identified. A master list linking participant identifying information with study IDs will be kept and stored separately from the data. Upon study completion, data collected for the purpose of recruitment will be destroyed.

Password protected audio files, identified by an identification number, will be uploaded to an account on a secure website only accessible by the transcriptionist. During transcription of audio recordings, identifying information (e.g., names, addresses, etc) will not be transcribed. The de-identified transcribed files will be encrypted and returned to the principal investigator using this secure account.
Participants will be informed that there is a possibility that their words may be cited verbatim in publications, presentations, and/or scientific meetings, that their identity will be kept confidential

and that we will remove any information that may reveal their identity. Participants will be given the option to refuse the use of their words being used and quoted verbatim in publications, presentations, and/or scientific meetings and will be asked to speak to the study team prior to signing the consent form.

Participants will be informed that audio or video recording will not be permitted except by the research team. They will be asked to not record or take pictures while taking part in the study or share or stream any part of the session, including on social media. Participants will also be asked to ensure that they are in a private place when taking part in the study, where their conversation cannot be overheard.

ii) Indicate how long personal health information will be retained in an identifiable form and why.

Audio recordings may have names and other identifying information. During transcription, identifying information (e.g. names, addresses, telephone numbers) will not be transcribed. Audio recordings will be deleted when the study is complete

iii) Who will have access to these data in the future.

Members of the study team.

I. Describe how and when the personal health information will be disposed of or returned to the health information custodian.
(Max ¼ page)

Electronic copies of the master list, consent forms and all de-identified study documents will be kept for a period of 7 years. Following this, all hard copies will be destroyed using cross-cut shredding. Electronic files will be deleted from the secure network at the end of the retention period

J. Has the investigator applied for approval to another REB? ☐ Yes ☒ No
If yes, provide the response to or status of the application.

K. Describe whether the investigators' interest in the disclosure of the personal health information or the performance of the research would likely result in an actual or perceived conflict of interest with other duties of the researcher.
Not Applicable ☒

L. Describe the anticipated public or scientific benefit of this study.

A physical activity intervention for individuals with dysvascular LLA may help to promote physical activity within this population.
