# Supplementary material for: A behaviour change intervention promoting physical activity following dysvascular amputation: Protocol for a pilot study
Source: PLoS One. 2025 Jul 10;20(7):e0326761. doi: 10.1371/journal.pone.0326761 (PMC12244559; doi:10.1371/journal.pone.0326761)
Supplement: S2 File — (PDF) [file pone.0326761.s002.docx]

Title: A Behaviour Change Intervention Promoting Physical Activity Following Dysvascular Amputation: A Pilot Study

**Principal Investigator:**

Dr. Crystal MacKay, Scientist, West Park Healthcare Centre; [crystal.mackay@westpark.org](mailto:crystal.mackay@westpark.org)

**Investigators:**

Dr. William C Miller, Professor, Department of Occupational Science & Occupational Therapy, UBC

Dr. Diana Zidarov, Scientist, Université de Montréal

Dr. Brian Chan, Affiliate Scientist, Toronto Rehabilitation Institute-UHN

Dr. Steven Dilkas, Physiatrist, West Park Healthcare Centre

Dr. Sander Hitzig, Senior Scientist, Sunnybrook Research Institute

Dr. Andresa Marinho Buzelli, Research Associate, Sunnybrook Research Institute

Dr. Amanda Mayo, Physiatrist, Sunnybrook Research Institute

Dr. Michael Payne, Physiatrist, University of Western Ontario

Dr. Amy Schneeberg, Statistician, Amy Schneeberg Statistical and Methods Consulting

Dr. Julia Totosy de Zepetnek, Associate Professor, University of Regina

Dr. Dalton Wolfe, Scientist, Lawson Health Research Institute

Dr. José Zariffa, Senior Scientist, University Health Network

Dr. Audrey Zucker-Levin, Professor, University of Saskatchewan

Dr. Susan Jaglal, Professor, University of Toronto

Brittany Pousett, Certified Prosthetist/Head of Research, Barber Prosthetics

Dr. Heather Underwood, Physiatrist, GF Strong Rehabilitation Centre

Table of Contents

| **Study Summary** | 3 |
| --- | --- |
| **Introduction** | 3 |
| Overview | 3 |
| Background | 3 |
| Study Objectives | 5 |
| **Methods** | 5 |
| Study Design | 5 |
| Participants | 6 |
| Experimental Intervention | 6 |
| Outcome Measures | 7 |
| Sample size | 10 |
| Recruitment | 11 |
| Group Allocation | 11 |
| Allocation Concealment | 12 |
| Masking | 12 |
| Qualitative Interviews | 12 |
| Data Analysis | 12 |
| Consent Process | 14 |
| Honorarium | 14 |
| Data Storage and Retention | 14 |
| Privacy and Confidentiality | 14 |
| Personal Health Information | 15 |
| Benefits and Risks | 15 |
| Burden | 15 |
| Clinical Relevance | 16 |
| Knowledge Translation Activities | 16 |
| References | 18 |

**Summary**

Loss of a lower limb due to diabetes can have a devastating impact on physical and mental health and quality of life. Individuals are at risk of other diseases such as cardiovascular disease, loss of the other limb and death. Physical activity can reduce risk of chronic disease and improve health outcomes; however, physical activity levels in people with limb loss are low, often due to reduced balance and walking ability. Rehabilitation services are not readily available and the cost of delivering such programs remains high. Working with individuals with lower limb amputation, we created a virtual, peer-led physical activity behaviour change intervention called IMproving Physical Activity through Coaching and Technology following Lower Limb Loss (IMPACT-L3). We will conduct a pilot study to assess feasibility and optimize design of a future trial of effectiveness.

1.0 Introduction

1.1 Overview

Diabetes-related lower limb amputation (LLA) is a leading cause of disability globally.^1^ In the US alone, 185,000 LLAs are performed annually and the number of LLAs are projected to double by 2050 as a result of increasing rates of diabetes and the aging population.^2^ In Canada, 7,300 people have a LLA each year.^3^ More than 80% of these amputations result from complications of diabetes and/or peripheral vascular diseases (dysvascular LLAs).^3^ While dysvascular LLA commonly affects adults over age 65, there has been a resurgence of diabetes-related amputations in younger and middle-aged adults.^4^ LLA is a significant life event which impacts mobility, mental health and ultimately quality of life.^5-10^ In addition to disability resulting from LLA, individuals with dysvascular LLA often have multimorbidity (mean of five health conditions)^11 12^ and 37% of individuals require a contralateral or revision amputation within 5 years which can further impact their function and quality of life.^13^ Moreover, this population has a poor survival rate, estimated between 17 months and 4 years ^14-18^ with cardiovascular comorbidities cited as the leading cause of death.^18-25^ Due to their complex health challenges, people with dysvascular LLA have high rates of health care utilization (50% readmission rate)^26^ leading to high healthcare costs. While Canadian cost estimates are unavailable, healthcare costs for amputation in the US are >4.3 billion annually.^27^ With such high costs, rehabilitation services for people with LLA are often limited.^28 29^ In Canada, only 18% of adults with LLA receive inpatient rehabilitation and services are more available in urban than remote areas.^30^ These findings underscore the need for accessible, low-cost approaches to optimize health, social participation and quality of life in this population.

There is strong evidence that adequate physical activity reduces risk of chronic disease, all-cause and cardiovascular mortality and improves quality of life.^31^ Moreover, physical activity is a cornerstone of management of LLA^32^ and may confer additional benefits for people with dysvascular LLA such as improving diabetes outcomes and prevention and management of cardiovascular disease.^31 33^ Unfortunately, dysvascular LLA is characterized by high disability and low physical activity levels which may be a consequence of the amputation and comorbid conditions.^36^ ^39^ ^41^ People with dysvascular LLA have reduced strength, balance, cardiorespiratory fitness, walking ability and increased energy expenditure during ambulation.^35^ ^38^ ^53^ ^54^ Consequently, they have impaired mobility leading to sedentary behaviour. The presence of comorbidities and secondary conditions including skin problems (e.g., pressure ulcers)^55-58^ and musculoskeletal conditions (e.g., low back pain) can further impact individuals physical activity levels.^59^ Across studies examining physical activity in people with dysvascular LLA, step counts ranged from 1250 steps/day in older prosthesis users to 3809±2189 steps/day in people with diabetes-related LLA.^40^ ^60-62^ People with dysvascular LLA accumulated 24±41 minutes per week of moderate to vigorous physical activity, well below the minimum recommended 150 minutes per week.^40^ Importantly, not everyone with LLA can use a prosthesis due to muscle weakness, cognitive impairment or skin problems.^63^ ^64^ It can be difficult for wheelchair users to accrue adequate physical activity.^65^

Given the limited availability of rehabilitation services for LLA, accessible interventions to support physical activity in people with LLA are needed. Evidence suggests that multifaceted interventions that incorporate social support and behaviour change techniques increase physical activity in older adults and people with chronic illness.^34-37^ In a national meeting, stakeholders ranked research on peer led physical activity programs for people with dysvascular LLA as a top research priority.^38^ To date, no peer-led physical activity interventions for people with dysvascular LLA have been evaluated.^39 40^ To address this gap, we co-created a novel physical activity behaviour change intervention comprised of peer coaching, wearable technology and education. Prior to conducting a trial to determine effectiveness of the intervention, a pilot study is required to assess feasibility and optimize design of a future trial.

**1.2 Background**

***1.2.1 Barriers to Physical Activity***

A multitude of barriers can restrict physical activity participation in people with LLA. These barriers include lack of knowledge of physical activity, transportation to get to spaces to participate in physical activity, geographic features of the environment (e.g., stairs), co-morbid conditions, and availability of programs.^41^ ^68^ Consistently across studies, individuals with LLA identified support from others to engage in physical activity as important, including follow-up phone calls and counselling.^41^ In prior research, social support from peers with limb loss was perceived as a facilitator of physical activity.^69^ These findings underscore the need for supportive physical activity interventions that are accessible. Addressing factors known to optimize physical activity (e.g. self-efficacy, knowledge, motivation, social support) will be important to improve participation in physical activity after dysvascular LLA.^41^

***1.2.2 Effects of Physical Activity Interventions: Systematic Reviews and Recent Trials***

The physiological, psychological, and social benefits of physical activity are well established.^31^ Even minimal amounts of physical activity can improve functioning and slow the effects of deconditioning that are associated with disability.^41^ A systematic review and meta-analysis examined the effects of exercise programs on health in individuals with LLA, including data from 10 randomized controlled trials (with data up to 2019).^39^ Overall, combined exercise programs (e.g., strength, balance, aerobic training) had positive effects on distance walked, cardiorespiratory fitness, muscle strength and function. However, **only four of the 10 studies included people with dysvascular LLA**.^42-45^ A more recent systematic review found that exercise performed one to three times per week improved balance, walking speed, walking endurance and transfer ability in adults with LLA, especially when combining aerobic exercises with lower limb strengthening or balance exercises.^46^ Similarly, few individuals with dysvascular LLA were included in the studies (35%).^46^ Two of the studies focused on behaviour change. One study was a pilot RCT, which examined the preliminary efficacy of a physical activity intervention in people with dysvascular LLA.^42^ The intervention included weekly 30-minute telephone sessions with a therapist for 12 weeks focused on health behavior change. Step count increased 1135 steps per day in the intervention group compared to 144 steps per day in the control group (P=.03). However, the sample was recruited from 3 hospitals and the sample was predominantly male (3/38 females) limiting generalizability of the findings. A similar feasibility study of a biobehavioral intervention including 12 video sessions delivered by a therapist to support physical activity behaviour change, established the feasibility of the intervention (90% retention rate, low safety risk).^47^ The study was limited by a small sample size of male veterans from one centre. To our knowledge, no studies have assessed a peer-led physical activity behaviour change intervention for people with dysvascular LLA.

***1.2.3 Co-Creation of a Physical Activity Behaviour Change Intervention***

In 2020, stakeholders (i.e., people with LLA, clinicians, researchers and community agencies) from across Canada identified research priorities on physical activity for people with LLA.^47^ Lack of opportunities for peer support for adults with dysvascular LLA was the second highest ranking issue. To advance a solution, participants outlined a plan to develop and evaluate an evidence-based physical activity program using trained peers. In addition, our team conducted a qualitative study exploring physical activity in people with LLA.^69^ Barriers to physical activity included access to physical activity programs for people with LLA (i.e., lack of availability, physical accessibility, and transportation). Social support from peers who provide encouragement and accountability was perceived to facilitate participation in physical activity.^69^ Building on this research and using prior literature and theory, our team of researchers, clinicians and people with LLA designed a physical activity behaviour change intervention: **IM**proving **P**hysical **A**ctivity through **C**oaching and **T**echnology following **L**ower **L**imb **L**oss (**IMPACT-L3**). A logic model for the intervention can be found in Appendix A.

**2.0 Study Objectives**

The **primary aim** is to assess the feasibility of conducting a randomized controlled trial to determine the effectiveness of a virtual peer-led physical activity intervention on levels of physical activity and self-efficacy compared to a wait-list control in people with dysvascular LLA.

**Specific objectives** are to:

1. Evaluate feasibility according to indicators of process, resources, management and treatment.
2. Explore perceptions of program characteristics, program implementation and study procedures among individuals with LLA; and
3. Explore the perceived feasibility and acceptability of the program among peer coaches.
4. Inform a sample size calculation for a future trial of effectiveness.
5. Assess construct validity of physical activity and self-efficacy measures among individuals with dysvascular LLA.

3.0 Methods

3.1 Study Design

This pilot study is a parallel group RCT with an embedded qualitative component. A pilot study is a subset of feasibility studies which asks questions about feasibility (whether the future trial can be done and, if so, how) but with a key design feature: in the pilot study, the future RCT is conducted on a smaller scale. Prospective recruitment, concealed group allocation, evaluator masked outcome evaluation and waitlist control will be employed. The research is guided by the MRC framework for evaluating complex interventions.^51^ We will follow the extension to the Consolidated Standards of Reporting Trials Statement for reporting pilot and feasibility RCTs.^87^

3.2 Participants

Individuals who meet the following inclusion criteria will be included: 1) Dysvascular LLA (LLA due to diabetes or vascular disease); 2) major LLA (at the ankle or above); 3) living in the community; 4) adult at least 18 years of age; 5)comfortable communicating in English and **a**ble to understand basic English; and 6) receptive to using a phone or tablet (e.g., to enable peer coaching, access to modules. Participants may be ambulatory with a prosthesis and/or use a wheelchair.

Exclusion criteria include: 1) actively receiving rehabilitation services related to physical activity/mobility; 2) recommended medical supervision for physical activity by health care provider, ^102^ or skin problems preventing usual activity and 3) not able to provide informed consent.

Participants who are interested in taking part in the study but who do not have access to a device will be offered the use of a device to borrow. The number of participants who may be offered the use of a device may be limited. Participants who may require additional support with the use of technology will be provided such supports to increase accessibility. This support may include instructions and support with accessing and signing in to Zoom/WebEx/Teams and walking through the use of Zoom/WebEx/Teams to facilitate peer coaching sessions and/or accessing and walking through the online modules and/or study measures.

**3.3 Experimental Intervention (IMPACT-L3)**

Theory-based interventions are more effective in increasing physical activity.^48^ Two theories provide a framework for IMPACT-L3. One is social cognitive theory, which is a useful theoretical lens for incorporating self-efficacy into interventions.^49 50^ Self-efficacy is the belief a person has in his or her ability to perform a behavior successfully.^51^ Self-efficacy is informed by skill mastery, vicarious experience, verbal persuasion, and reinterpretation of physiological and affective symptoms.^51^ The other is self-determination theory, which provides a framework for cultivating an autonomy-supportive social environment that promotes behavior change.^52^ This is achieved by satisfying three basic psychological needs of autonomy (i.e., volition in one’s own behavior), competence (i.e., interacting effectively with one’s environment by mastering tasks), and relatedness (i.e., sense of belonging).^52^ These constructs were integrated into IMPACT-L3. As eHealth solutions have the potential to increase access and improve health outcomes,^53 54^ IMPACT-L3 will be delivered entirely virtually.

Peer health coaching (~30 minutes weekly) will be delivered by a peer trainer over 8 weeks. Peer trainers, who have experienced a dysvascular LLA themselves, will be matched to a participant based on gender and level of amputation to promote a sense of understanding and belonging. Individuals with higher level amputations (i.e., above knee) are less physically active and face more challenges with mobility (e.g., greater energy expenditure)^55 56^ and may benefit from a peer with similar experience. Peers will be trained to implement the intervention including specialized training in brief action planning (BAP), a structured support technique grounded in the principals and practices of motivational interviewing (Appendix C). Training will be delivered by an organization which provides certified training in BAP ([Centre for Collaboration, Motivation and Innovation](https://ccmi.learnupon.com/store/300131-brief-action-planning-certification))^57^ including an online course and practice and feedback. The trained peer will help participants **set goals and create an action plan for physical activity** that they feel confident that they can achieve. During weekly meetings, peers will help participants problem solve challenges to physical activity and overcome barriers. The peer trainer will deliver the intervention through **voice or video calls** (depending on preference). Video calls will be preferred because “face-to-face” interactions may reinforce vicarious experiences (e.g., peers demonstrate movements). Peers will complete a standardized form at every interaction to review goals, goal progression (e.g., Apple Watch data), action plans, and document barriers/facilitators. Peer health coaches will be asked to complete a standard form for each coaching session (Appendix D). Weekly debriefings among peers and the research team will problem solve challenges and monitor peer burden.

To improve competence and skills to enhance physical activity, participants will have access to five web-based modules developed based on qualitative interviews and co-design workshops with people with LLA. Modules include content on physical activity including benefits of physical activity, types of physical activity (strengthening, aerobic, balance, flexibility), intensity of activity (light, moderate, vigorous), exercise safety and limb management to enable physical activity, and recommended physical activity. The written materials that correspond with the online modules is included in Appendix E. The modules will be housed on a learning management system at the University of Toronto. They will include videos. Participants will be given a login and will be asked to review modules at their own pace each week for the first 5 weeks.

To support behaviour change, participants will be provided with a wearable to track their physical activity: an Apple Watch to be worn at the wrist of the non-dominant side. An off the shelf wearable was selected for the study **to optimize sustainability of the intervention in a real-world setting**. Apple Watch has been shown to be accurate in tracking step count in a subgroup of populations^58^ and can measure wheelchair pushing thus making it the best option available.^59^ Participants will be trained to wear and use the Apple Watch 24 hours a day during the intervention time only, including water-based activity. Wearables that provide personalized, and actionable feedback promote better behavior change outcomes.^60^  Data from the Apple Watch will be shared verbally with the peer during discussions between participants and peers during the weekly virtual sessions to facilitate behaviour change.

**Control:** The control group will continue with their usual health care and be offered the intervention program at the end of the follow-up period (wait-list control).

**3.4 Outcome Measures**

Data will be collected at baseline, week 9 and a short follow-up 4 weeks later (3 months).

**3.4.1 Feasibility Measures**

The primary outcome of this study is feasibility of implementing the intervention and conducting the trial. Feasibility indicators will be collected including assessment of process, resource, management and treatment indicators (Appendix D).

**Process Indicators:**

*Recruitment rate* will be evaluated by the number and proportion of participants recruited per month and the number and proportion of peers recruited.

*Consent rate* will be calculated as the percent of recruited individuals who provide consent per month.

*Withdrawal rate* will be calculated as the percent of study participants withdrawing by week 9 (T2) and 3 months (T3).

*Acceptability* will be assessed in qualitative interviews and by the theoretical framework of acceptability (TFA),^103^ a brief questionnaire developed to assess acceptability in the design, evaluation and implementation of interventions.

**Resource Indicators:**

*Participant adherence* will be measured as the percentage of peer coaching sessions participants attend. Participants’ usage of web-based modules will be measured (i.e., number of completed modules, number of logins).

*Peer coach adherence* will be assessed by tracking the total number of peer coaching sessions attended by the peer-trainer.

*Participant and tester burden* will be measured by the amount of time it took to administer study outcomes at T1, T2, and T3 and the acceptability of the evaluation time commitment from the perspective of participants.

*Feasibility of data collection* will be evaluated as the percentage of participants with complete data on each measure at each evaluation time point. For accelerometers, the percentage of devices that were returned at baseline and follow-up (9 weeks, 3 months) and the amount of valid wear time will be assessed.

**Management Indicators:**

*Participant processing time* will be assessed as time from initial contact to enrolment.

*Intervention fidelity* will focus on adherent and competent delivery of the intervention. It will be evaluated using the study-specific checklist outlining key components of the intervention completed by peers. A subset of peer coaching sessions (at least one per dyad) will be recorded and reviewed by research staff using the checklist.

**Treatment Indicators**:

*Adverse events* will be measured as the number of adverse events that occurred during physical activity for the intervention. Adverse events (e.g., falls) will be documented by peers on the standardized form used at each coaching session.

**3.4.2 Proposed primary outcomes for the trial (Appendix D)**

While this pilot trial is not powered to detect meaningful differences in these outcomes, collecting data on our proposed outcomes can assess and ensure that there are no issues with the collection and completion of these measures in preparation for a future trial.

**Objective Physical Activity (Accelerometer).** A tri-axial accelerometer is a lightweight device used to measure total activity counts. The total volume of physical activity (activity counts) will be measured. This measure has the advantage of integrating the frequency, intensity, and duration of movement and combining them into an overall measure of physical activity.^61^ The sum of the total count for the day will be used and averaged over the measurement period. Total activity counts can be a better metric than the number of minutes per day spent in various physical activity intensity categories because it incorporates all levels of intensity. Recent evidence suggests that light, moderate and vigorous activity **all** have health benefits.^62^ ^63^ Ambulatory participants will wear the monitor on a waist belt on the side of the shortest residual limb which provides the most valid data in people with LLA.^64^ Wheelchair users will wear ActiGraph™ wGT3X-BT on the non-dominant arm.^65^ For wheelchair users a second ActiGraph™ placed on the rear wheel is recommended (waterproof box installed on the rear wheel using tie wraps). Participants will wear the accelerometer at all times except while bathing or swimming for a period of 7 days pre- and post-intervention and at 3-month follow-up. Only data from days in which participants wear the activity monitors for >10 hours per day will be included in the analyses as per previous approaches, including individuals with dysvascular amputation.^42^

**Self-efficacy for Exercise Scale.** A self-report measure (9 items) that includes situations that may influence physical activity participation. Participants will respond to each item on a 0 (not very confident) to 10 (very confident) scale. This is a valid measure of exercise self-efficacy in older adults.^66^

**3.4.3 Secondary outcomes (Appendix D)**

**Self Efficacy for Exercise – Dysvascular Lower Limb Amputees.** 4-item measure will be administered in addition the standardized self-efficacy for exercise measure and self-regulatory efficacy for exercise measure to address contextual factors relevant to dysvascular lower limb amputees. Items are informed by a meta synthesis of data from dysvascular lower limb amputees as well as recommendations from the literature for measuring self-efficacy.^79,83^ These items represent physical activity challenges reported by this subgroup.

**Self-Regulatory Efficacy for Exercise.** 8-item measure to assess participants’ confidence in their ability to manage their exercise. The measure pertains to behaviours necessary to self-regulate exercise over the next 4 weeks, such as scheduling exercise, planning exercise sessions, overcoming barriers that may interfere with exercise, and preventing relapse by overcoming temporary exercise lapses. Items are assessed using a confidence scale ranging from 0 per cent (not at all confident) to 100 per cent (completely confident) and in accordance with recommendations in the literature.^51, 80, 81^ These items have been used previously in exercise research, in which internal consistencies ranged from .84 to .93 (e.g., Woodgate and Brawley ^67^).

**Center for Epidemiologic Studies Depression Scale (CES-D).** The CES-D will assess participant's self-rated depressive symptoms. It is a 20-item scale that refers to symptoms in the last week. The sum of the 20 items provides a score ranging from 0-60 with higher scores indicating greater symptoms. The CES-D shows good internal consistency (Cronbach's alpha coefficient of 0.85).^68^ The CES-D is the best supported measure for diabetes^68^ and has been used in RCTs with patients with LLA.

**The Physical Activity Scale for Individuals with Physical Disability (PASIPD).** The PASIPD is a 13-item questionnaire with subscales measuring leisure time, household and work-related activities.^69^ The PASIPD has demonstrated reliability and validity in people with physical disabilities.^70^

**Leisure-Time Physical Activity for People with Disabilities Questionnaire (LTPAQ-D).** The LTPAQ-D is a self-report measure that assesses minutes of mild-, moderate-, and vigorous-intensity leisure time PA (i.e., activity that requires physical exertion and that one chooses to do in their free time performed over the past 7 days. Support for the LTPAQ’s criterion validity and test–retest reliability has been demonstrated in people with spinal cord injury and other disabilities^71^.

**Activities-specific Balance Confidence scale (ABC).** ABC is a self-report measure used to assess perceived balance confidence. The total score varies from 0 to 100, with higher scores indicating more confidence. Validity and test-retest reliability (ICC=0.91) have been shown for people with LLAs.^72^

**Prosthesis Evaluation Questionnaire - Mobility Section** (PEQ-MS). The PEQ-MS score will be used to measure the amount of difficulty completing locomotion tasks. The PEQ-MS is a reliable (ICC: 0.0.73-0.90) and internally consistent (Cronbach alpha = 0.96) measure in people with LLA.^73 74^

#### **Short Form Health Survey 12 Item survey (SF-12).** The SF-12 will be used to measure health related quality of life. Twelve items are categorized into a physical and a mental domain; the scores of the quality of life domains range from 0 to 100, with higher scores referring to higher quality of life. Several studies have reported good psychometric properties of the SF-12 including studies in older adults.^75^

**Frenchay Activities Index (FAI).** The 15-item FAI captures information on social activities. The items reflect the frequency of performance of basic and instrumental activities of daily living over three domains. The total scores range from 0 (very limited) to 45 (very active). Research demonstrates that this index is reliable and valid for use in individuals with LLA.^76^

***Descriptive Variables and Covariates:*** Demographic characteristics will be collected by questionnaire (Appendix D for participant demographics and peer demographics/profile). Participants will be asked about sex at birth (male, female or intersex), and what best describes their current gender identity (woman, man, non-binary etc). Age, employment status, education level, personal/household income, marital status, and living arrangement will also be collected. LLA characteristics will be collected: time since amputation, cause of amputation, level of amputation, co-morbid conditions, primary mode of mobility, mobility level (measured by the Amputee Single Item Mobility Measure^77^; Appendix D), cognition (measured by the Montreal Cognitive Assessment; Appendix B^78^), which will be administered by research staff, and previous participation in rehabilitation. Self-reported healthcare use (Appendix D) during the intervention will be documented.

**3.4.4 Data Collection Methods:**

All participants will be asked to complete the online questionnaires described above via REDCap. Participants will be provided with a link by email. They will be provided with the Consent Form (Appendix H), which they will be asked to read and sign before proceeding with the questionnaire. Individuals who are not able to complete the questionnaires via REDCap will be given an option to receive the questionnaire via email or by mail. To increase response rates, a research coordinator will book a time with the participant to support completion of the questionnaires (the participant will self-complete the questionnaire in REDCap; the research staff will be available to support any technical challenges). The ActiGraphTM activity monitor will be mailed to the participant with instructions on how to use it to measure total physical activity counts per day. A research coordinator will be available to support the participant via phone or Zoom/WebEx/Teams. Peer Health coaches will be provided with the Consent Form (Appendix H), which they will be asked to read and sign before proceeding with the demographics questionnaire (Appendix D), PASIPD (Appendix D) and one-on-one qualitative interview described below.

3.5 Sample Size

Since this is a pilot study, a formal sample size calculation was not performed. The recruitment of 30 participants per arm of the trial is judged to be feasible and will produce a robust amount of data while piloting across provinces in English and French (allowing a 20% drop out). This is consistent with Whitehead et. al.’s suggestions that for a trial designed with 90% power and two- sided 5% significance, pilot trial sample sizes for each treatment arm should be 25 and 15 for standardized effect sizes that are small (0.2) and medium (0.5) respectively.^84^

3.6 Recruitment

Potential participants will self-identify or be identified by a member of the research team or health professional. Individuals with dysvascular LLA will be recruited from rehabilitation hospitals with support from collaborators. Patients who have completed rehabilitation services as an inpatient or outpatient following LLA often continue to have long term follow-up appointments with the clinical team (e.g., annual visit, assessment of prosthesis, etc). Participants will be recruited from rehab clinics in the community, for example, prosthetics clinics, as well as primary healthcare clinics. Potential participants will also be recruited through community centers (e.g., Variety Village) and the networks of the research team. A research database of individuals who have indicated in past research their interest to take part in future studies will be used to identify potential participants. Individuals who have consented to being contacted for future study participation may receive a Study Invitation Letter (Appendix F).

Study posters (Appendix F for recruitment materials) will be used to recruit potential participants. Study posters will be displayed in the clinical practice areas and in outpatient amputation clinics at West Park Healthcare Centre, at rehabilitation centres/hospitals, at other clinic sites that treat individuals with a LLA (e.g., St John’s Rehab), patient organizations, community programs and organizations (e.g., Variety Village), on amputation and/or limb loss websites (i.e., Amputee Coalition of Canada), social media accounts of partners including but not limited to Amputee Coalition of Canada, newsletters, listserv/contact listservs of affiliated organizations and networks of the research team across Canada.

Social media advertisements (Appendix F) will be used to recruit potential participants. Social media ads will be displayed on West Park social media and websites (e.g. Twitter, Facebook, WP website) and other hospital, health centre, or affiliated organizations’ social media accounts and websites.

Study information sheets and study invitation letters (Appendix F) will be used to recruit potential participants. Clinicians at the rehabilitation hospitals will be informed about the study via a Study Information Sheet explaining the project. Study Invitation Letter will be provided to potential participants.

A research coordinator will have initial contact with potential participants. Individuals who self-identify and contact the study team via phone or email will receive a response from the research coordinator who will review the study information and determine their study eligibility (Appendix B for screening documents). Potential participants may have initial contact with a health professional within their circle of care or a research coordinator or administrator during an in person or virtual clinic visit. They may receive a Study Invitation Letter and may be asked by clinical or administrative staff if they agree to being contacted by a research coordinator to discuss the study.

3.7 Group Allocation

Participants will be assigned to one of two groups: intervention group and control group.

Randomization stratified by age and sex will be employed with permuted block randomization of varying block size (2 and 4) to reduce the possibility of selection bias. The allocation schedule will be created using an online tool by a person outside of the research team who is not involved in recruitment. Age is a predictor of physical activity^85^ and could be associated with the efficacy of the intervention. The age groupings (>65, 65+) are consistent with research demonstrating differences in physical activity by age in people with LLA.^101^ Stratification will be employed to ensure representation across age ranges and sex to enable investigation of these variables as effect modifiers.

An independent statistician will provide the randomization list through REDCap (REDCap Software, Vanderbilt University and National Institute of Health, USA). Upon enrolment, a study ID number will be allocated to each participant. The study ID number will be linked to the randomization list.

**3.8 Allocation concealment**

After completing the baseline assessment, the research coordinator will reveal the group allocation of the participant through the REDCap randomization list.

3.9 Masking

Due to the nature of the intervention, masking to receipt (or not) of the intervention is impossible. In this study, one assessor will collect all data. They will be masked to group allocation. We will ask participants not to disclose their group allocation during assessments.

3.10 Qualitative Interviews

For **objectives 2 and 3**, we will use a qualitative descriptive approach ^79^ to understand participants’ and peers’ experiences with IMPACT-L3. The research will be situated within an interpretive research paradigm.^80^ This component will be critical to improving the intervention and refining the protocol for the main trial. To explore perceptions of recruitment approaches, data collection procedures and measures, time burden and acceptability of program implementation (**Objective 2**), one-on-one semi-structured telephone or Zoom/WebEx/Teams interviews (Interview guides for peer health coaches and people with limb loss: Appendix G) lasting ~45-60 minutes will be conducted with participants after completion of the intervention. Participants will be asked to share their perceptions of program characteristics. For example, participants will be asked about their perceptions of Apple Watch as a tool to support behaviour change including the appropriateness of the data provided (e.g., distance) and the usability of the device. Findings will inform refinements of the intervention, **if needed** (e.g., development of a custom app to use with Apple Watch). Data will be collected until data saturation. Based on prior research^81^, we anticipate recruiting ~15-20 participants. We will sample purposively for variation in age, gender and level of amputation. **For objective 3**, we will conduct semi-structured interviews with all consenting peers to understand their experiences and identify considerations for optimizing the intervention.

**3.11 Data Analysis**

**RCT:** Descriptive statistics (mean, standard deviation, counts (percentage)) will be used to summarize continuous and categorical data, as appropriate. Questionnaire data will be analyzed in Excel and/or SPSS.

**Objective 1:** Descriptive statistics (mean, standard deviation, counts (percentage)) will be used to summarize continuous and categorical data, as appropriate. **Progression criteria:** Mellor et al. (2023)^86^ recommend using guidelines rather than rules when using progression criteria in a feasibility study, avoiding consideration of one indicator in isolation, and involvement of partners in the decision making. These principles, with the RAG (red amber green) or traffic light approach, will be used to guide the research team in determining what changes may be required to address identified challenges based on the indicators and in determining the feasibility of progression to a trial. See Table, Appendix D for specifics.

**Objective 4:** As a pilot study the analysis will be mainly descriptive. We will use the data from this pilot study to summarize overall and by group to help inform outcomes measures and sample size estimation for the main trial. The baseline activity, health and demographic characteristics and all other covariates for the intervention and control groups will be described by frequencies and percentages and mean and standard deviation as appropriate. The proposed primary and secondary outcomes will be described by mean, median, standard deviation and interquartile range. They will be visualized with box and whisker plots and histograms by intervention group and time point. Descriptive statistics and estimation, using confidence intervals, will be used to inform sample size estimation. The confidence interval will be interpreted with consideration of clinical relevance. If sufficient data, the relationship between intervention/control and the primary and secondary outcomes will be examined using linear mixed effects models including participant ID as a random effect and the observations taken at 9 weeks and 3 months as the outcome while controlling for the baseline observation. Based on stratification of sex and age in randomization, both of these variables will also be included in the models as covariates.

**Objective 5:** Cross sectional data from baseline will be used to assess construct validity of the self-efficacy for exercise questionnaire in people with dysvascular LLA and LTPAQ-D in people with dysvascular LLA. Pearson's correlation coefficients and Spearman's correlation coefficients will be used as appropriate.

We hypothesized the following associations in terms of magnitude and direction: a) a moderate positive correlation between self-efficacy for exercise, self-regulatory efficacy for exercise**,** mobility (Prosthesis Evaluation Questionnaire - Mobility Section), balance confidence (ABC), and health-related quality of life (SF-12). b) A negative correlation between self-efficacy for exercise and depressive symptoms (CES-D).

We hypothesized the following associations in terms of magnitude and direction: a) a moderate positive correlation between the LDPAQ-D and the PASIPD questionnaire, mobility (Prosthesis Evaluation Questionnaire - Mobility Section), and balance confidence (ABC).

**Qualitative Interviews:** Inductive thematic analysis will be employed to analyze qualitative interviews.^125^ NVivo software will be used for qualitative analysis. Team members will independently code four transcripts to develop a preliminary coding framework. The coding framework will be applied to four transcripts; team members will review the coding and modifications will be made to the coding framework. Codes will then be organized into categories or themes to explain the data.

**3.12 Consent Process**

Individuals who agree to participate and are eligible will have the opportunity to ask questions before reviewing and signing the Consent Form (Appendix H for consent forms). Participants will sign a Consent Form prior to taking part. Consent Forms will be signed and returned online via REDcap. Individuals who are not able to sign the Consent Form via REDCap will be given an option to sign and return the Consent Form in person, via email or by mail with a prestamped and addressed envelope. Potential participants will be given as much time as they need to decide if they would like to participate, up until the time that the study activities are scheduled to occur.

Upon obtaining consent from participants, the research coordinator will schedule the first online meeting with a blinded assessor to complete the baseline assessment. The research coordinator will log onto the online randomization system to determine the next allocation. The research coordinator will forward the participant’s contact information to schedule the first online meeting.

**3.13 Honorarium**

Participants will receive a gift certificate valued at $50 for each of the 3 assessments. Twenty participants and/or peer coaches will be asked to take part in a qualitative interview. All those who complete an interview will receive a gift certificate valued at $50.Participants will be able to keep the AppleWatch at the end of the study.

**3.14 Data Storage and Retention**

Electronic copies of the master list, consent forms and all de-identified study documents will be kept for a period of 7 years. Following this, all hard copies will be destroyed using cross-cut shredding. Electronic files will be deleted from the secure network at the end of the retention period. Audio recordings will be deleted when the study is complete.

**3.15 Privacy and Confidentiality**

Data will be password protected and stored on the West Park Healthcare Centre network drive that has firewalls and security measures in place. Hard copy records will be stored in a locked cabinet in a secure location at West Park Healthcare Centre. Access to records and data will be limited to authorized persons.

Study data will be de-identified. A master list linking participant identifying information with study IDs will be kept and stored separately from the data. Upon study completion, data collected for the purpose of recruitment will be destroyed.

Password protected audio files, identified by an identification number, will be uploaded to an account on a secure website only accessible by the transcriptionist. During transcription of audio recordings, identifying information (e.g., names, addresses, etc) will not be transcribed. The de-identified transcribed files will be encrypted and returned to the principal investigator using this secure account.

Participants will be informed that there is a possibility that their words may be cited verbatim in publications, presentations, and/or scientific meetings, that their identity will be kept confidential and that we will remove any information that may reveal their identity. Participants will be given the option to refuse the use of their words being used and quoted verbatim in publications, presentations, and/or scientific meetings and will be asked to speak to the study team prior to signing the consent form.

Participants will be informed that audio or video recording will not be permitted except by the research team. They will be asked to not record or take pictures while taking part in the study or share or stream any part of the session, including on social media. Participants will also be asked to ensure that they are in a private place when taking part in the study, where their conversation cannot be overheard.

**3.16 Personal Health Information**

Personal information (names and contact information) will only be used for administrative purposes (i.e., scheduling an interview). Demographic data will be used to describe the study population (e.g., mean age, number of male/female participants). Demographic data from individuals with amputations will include: date of birth (month and year), city of residence, country of birth, gender and sex, level of amputation, date and cause of amputation, date of admission and discharge, level of education, work status and living arrangements, comorbidities, primary mode of mobility, etc. (Appendix D).

**3.17 Benefits and Risks**

There are **minimal** additional risks to participants in the study, **which are inherent to physical activity participation**. However, the long-term benefits outweigh those risks. Telephone-based physical activity interventions have been shown to be safe for people with dysvascular LLA in RCTs. To enhance safety with physical activity (e.g. reduce risk of falls, skin problems), LLA-specific education modules will address exercise safety and peers will be trained in how to address risks.

During interviews, some participants may feel uncomfortable answering questions about their health. All participants will be informed they are not required to respond and may “pass” any questions they do not wish to address.

While the risk is low, participants could experience distress, for example, while completing assessments. If any participants exhibit signs of distress while completing the assessments, the research team will follow up with those participants and if needed, provide them with a list of mental health resources (Appendix I).

**3.18 Burden**

Participant time commitment include attending weekly 30-minute sessions for 8 weeks, which they will attend virtually. Participants will also complete 5 web-based instructional videos (modules) to be at their own pace which will take up to 30 minutes to complete. Participants will also complete 3 assessments lasting approximately one hour each. Peer health coaches will be asked to complete a standard form for each coaching session, which will take approximately 5-10 minutes. Post-intervention participants and peer coaches may complete a one-on-one semi-structured telephone or Zoom/WebEx/Teams interviews lasting ~45- 60 minutes.

3.19 Clinical Relevance

Individuals with LLA report low participation in PA and there is limited availability of rehabilitation services. Even minimal increases in PA can improve outcomes. Self-efficacy is a theory-informed cognitively behavioural variable linked with exercise, thereby changes in self-efficacy are expected to lead to positive exercise outcomes. We will pilot test a theory-informed intervention prior to a larger study testing the intervention in the future. This research is an important step towards supporting physical activity behaviour change in people with dysvasular LLA.

**3.20 Knowledge Translation Activities**

We have adopted an integrated KT approach. We will disseminate results through lay summaries, infographics, fact sheets, news stories, webinars, and relevant national (e.g., Ontario Association of Amputee Care conference) and international meetings (e.g., American Congress of Rehabilitation Medicine) to reach multiple stakeholders. Tailored messages to target specific audiences will be produced.

3.21 Operational Impact

A physical activity intervention for individuals with LLA may help to promote physical activity within this population. This project will produce a physical activity intervention for people with LLA.

3.22 Budget information

See Appendix J for budget justification.

3.23 Project Time Line

A project timeline is presented in Table 1 below.

Table 1: Project Timeline

| **Activity** | **Year 1** | | | | **Year 2** | | | |
| --- | --- | --- | --- | --- | --- | --- | --- | --- |
|  | **Q1** | **Q2** | **Q3** | **Q4** | **Q1** | **Q2** | **Q3** | **Q4** |
| ***Pre-Pilot Study Preparation*** |  |  |  |  |  |  |  |  |
| Submit REB applications | X |  |  |  |  |  |  |  |
| Peer training (CCMI) | X |  |  |  |  |  |  |  |
| Module development and finalizing | X |  |  |  |  |  |  |  |
| ***Pilot RCT*** |  |  |  |  |  |  |  |  |
| Recruitment and randomization |  | X | X | X |  |  |  |  |
| Quantitative data collection (T1, T2, T3) |  | X | X | X | X | X |  |  |
| Wait list controls offered intervention |  |  |  |  |  |  | X | X |
| ***Qualitative Data Collection and Analysis*** |  |  |  |  |  |  |  |  |
| Collect and analyze (iterative data collection and analysis) |  |  |  |  | X | X | X |  |
| ***Quantitative Analysis*** |  |  |  |  |  |  |  |  |
| Analysis of feasibility measures, primary and secondary outcomes (T1, T2, T3) |  |  |  |  |  |  | X | X |
| Refinement of IMPACT-L3 study protocol for full scale trial |  |  |  |  |  |  |  | X |
| ***Knowledge Translation*** |  |  |  |  | X | X | X | X |
| Peer-reviewed publications, conference presentations | X |  |  |  |  |  |  | X |
| Meetings with partners and collaborators, newsletters, news stories, webinars, briefing reports, lay summaries, infographics, fact sheets | X | X | X | X | X | X | X | X |
|  | | | | | | | | |

References

1. Lazzarini PA, Pacella RE, Armstrong DG, et al. Diabetes-related lower-extremity complications are a leading cause of the global burden of disability. Diabet Med 2018 doi: 10.1111/dme.13680 [published Online First: 2018/05/24]

2. Ziegler-Graham K, MacKenzie EJ, Ephraim PL, et al. Estimating the prevalence of limb loss in the United States: 2005 to 2050. Arch Phys Med Rehabil 2008;89(3):422-9. doi: 10.1016/j.apmr.2007.11.005 [published Online First: 2008/02/26]

3. Imam B, Miller WC, Finlayson HC, et al. Incidence of lower limb amputation in Canada. Can J Public Health 2017;108(4):e374-e80. doi: 10.17269/cjph.108.6093 [published Online First: 2017/11/10]

4. Geiss LS, Li Y, Hora I, et al. Resurgence of Diabetes-Related Nontraumatic Lower-Extremity Amputation in the Young and Middle-Aged Adult U.S. Population. Diabetes Care 2019;42(1):50-54. doi: 10.2337/dc18-1380 [published Online First: 2018/11/10]

5. Sinha R, Van Den Heuvel WJ. A systematic literature review of quality of life in lower limb amputees. Disabil Rehabil 2011;33(11):883-99. doi: 10.3109/09638288.2010.514646 [published Online First: 2010/09/10]

6. Pezzin LE, Dillingham TR, MacKenzie EJ. Rehabilitation and the long-term outcomes of persons with trauma-related amputations. Arch Phys Med Rehabil 2000;81(3):292-300. doi: 10.1016/s0003-9993(00)90074-1 [published Online First: 2000/03/21]

7. Graz H, D'Souza VK, Alderson DEC, et al. Diabetes-related amputations create considerable public health burden in the UK. Diabetes Res Clin Pract 2018;135:158-65. doi: 10.1016/j.diabres.2017.10.030 [published Online First: 2017/11/15]

8. Fortington LV, Rommers GM, Geertzen JH, et al. Mobility in elderly people with a lower limb amputation: a systematic review. J Am Med Dir Assoc 2012;13(4):319-25. doi: 10.1016/j.jamda.2010.12.097 [published Online First: 2011/04/01]

9. Fortington LV, Dijkstra PU, Bosmans JC, et al. Change in health-related quality of life in the first 18 months after lower limb amputation: a prospective, longitudinal study. J Rehabil Med 2013;45(6):587-94. doi: 10.2340/16501977-1146 [published Online First: 2013/04/30]

10. Amtmann D, Morgan SJ, Kim J, et al. Health-related profiles of people with lower limb loss. Arch Phys Med Rehabil 2015;96(8):1474-83. doi: 10.1016/j.apmr.2015.03.024 [published Online First: 2015/04/29]

11. Hong CCT, J. H.; Lim, S. H.; Nather, A. Multiple limb salvage attempts for diabetic foot infections: is it worth it? The bone & joint journal 2017;99-B(11):1502-07. doi: <https://dx.doi.org/10.1302/0301-620X.99B11.BJJ-2016-0793.R2>

12. Mayo AL, Viana R, Dilkas S, et al. Self-reported health condition severity and ambulation status postmajor dysvascular limb loss. Prosthetics and Orthotics International 2022;46(3):239-45. doi: 10.1097/pxr.0000000000000106

13. Liu R, Petersen BJ, Rothenberg GM, et al. Lower extremity reamputation in people with diabetes: a systematic review and meta-analysis. BMJ Open Diabetes Res Care 2021;9(1) doi: 10.1136/bmjdrc-2021-002325 [published Online First: 2021/06/12]

14. Ebskov LB. Diabetic amputation and long-term survival. Int J Rehabil Res 1998;21:403-8.

15. Pohjolainen T, Alaranta H. Ten-year survival of Finnish lower limb amputees. Prosthetics and Orthotics International 1998;22:10-6.

16. Tseng CH, Chong CK, Tseng CP, et al. Mortality, causes of death and associated risk factors in a cohort of diabetic patients after lower-extremity amputation: a 6.5-year follow-up study in Taiwan. Atherosclerosis 2008;197(1):111-7. doi: 10.1016/j.atherosclerosis.2007.02.011

17. Ebskov B. Relative mortality and long term survival for the non-diabetic lower limb amputee with vascular insufficiency. Prosthetics and Orthotics International 1999;23:209-16.

18. Stewart CPU, Jain AS. Cause of death of lower limb amputees. Prosthetics and Orthotics International 1992;16:129-32.

19. Kristensen MT, Holm G, Kirketerp-Moller K, et al. Very low survival rates after non-traumatic lower limb amputation in a consecutive series: what to do? Interactive cardiovascular and thoracic surgery 2012;14(5):543-7. doi: 10.1093/icvts/ivr075

20. Viswanathan V, Wadud JR, Madhavan S, et al. Comparison of post amputation outcome in patients with type 2 diabetes from specialized foot care centres in three developing countries. Diabetes research and clinical practice 2010;88(2):146-50. doi: 10.1016/j.diabres.2010.02.015

21. Ploeg AJ, Lardenoye JW, Vrancken Peeters MP, et al. Contemporary series of morbidity and mortality after lower limb amputation. European journal of vascular and endovascular surgery : the official journal of the European Society for Vascular Surgery 2005;29(6):633-7. doi: 10.1016/j.ejvs.2005.02.014

22. Inderbitzi R, Buettiker M, Enzler M. The long-term mobility and mortality of patients with peripheral arterial disease following bilateral amputation. European Journal of Vascular and Endovascular Surgery 2003;26(1):59-64. doi: 10.1053/ejvs.2002.1868

23. Thomas SRYW, Perkins JMT, Magee TR, et al. Transmetatarsal amputation: an 8-year experience. Ann R Coll Surg Engl 2001;83:164-6.

24. Peng CWB, Tan SG. Perioperative and rehabilitative outcomes after amputation for ischemic leg gangrene. Ann Acad Med Singapore 2000;29:168-72.

25. Modan M, Peles E, Halkin H, et al. Increased cardiovascular disease mortality rates in traumatic lower limb amputees. Am J Cardiol 1998;82:1242-7.

26. Kayssi A, de Mestral C, Forbes TL, et al. Predictors of hospital readmissions after lower extremity amputations in Canada. J Vasc Surg 2016;63(3):688-95. doi: 10.1016/j.jvs.2015.09.017 [published Online First: 2015/11/28]

27. Dillingham TR, Pezzin LE, Shore AD. Reamputation, mortality, and health care costs among persons with dysvascular lower-limb amputations. Arch Phys Med Rehabil 2005;86(3):480-6. doi: 10.1016/j.apmr.2004.06.072 [published Online First: 2005/03/11]

28. Dillingham TR, Pezzin LE, Mackenzie EJ. Discharge destination after dysvascular lower-limb amputations. Arch Phys Med Rehabil 2003;84(11):1662-8. doi: 10.1053/s0003-9993(03)00291-0 [published Online First: 2003/11/26]

29. Resnik LJ, Borgia ML. Factors associated with utilization of preoperative and postoperative rehabilitation services by patients with amputation in the VA system: an observational study. Phys Ther 2013;93(9):1197-210. doi: 10.2522/ptj.20120415 [published Online First: 2013/05/04]

30. Kayssi A, Dilkas S, Dance DL, et al. Rehabilitation Trends After Lower Extremity Amputations in Canada. PM R 2017;9(5):494-501. doi: 10.1016/j.pmrj.2016.09.009 [published Online First: 2016/09/25]

31. Warburton DER, Bredin SSD. Health benefits of physical activity: a systematic review of current systematic reviews. Curr Opin Cardiol 2017;32(5):541-56. doi: 10.1097/HCO.0000000000000437 [published Online First: 2017/07/15]

32. Webster JB, Crunkhorn A, Sall J, et al. Clinical Practice Guidelines for the Rehabilitation of Lower Limb Amputation: An Update from the Department of Veterans Affairs and Department of Defense. Am J Phys Med Rehabil 2019;98(9):820-29. doi: 10.1097/PHM.0000000000001213 [published Online First: 2019/08/17]

33. Diabetes Canada Clinical Practice Guidelines Expert C, Sigal RJ, Armstrong MJ, et al. Physical Activity and Diabetes. Can J Diabetes 2018;42 Suppl 1:S54-S63. doi: 10.1016/j.jcjd.2017.10.008 [published Online First: 2018/04/14]

34. Leidy NK, Kimel M, Ajagbe L, et al. Designing trials of behavioral interventions to increase physical activity in patients with COPD: insights from the chronic disease literature. Respir Med 2014;108(3):472-81. doi: 10.1016/j.rmed.2013.11.011 [published Online First: 2013/12/10]

35. Latimer-Cheung AE, Arbour-Nicitopoulos KP, Brawley LR, et al. Developing physical activity interventions for adults with spinal cord injury. Part 2: motivational counseling and peer-mediated interventions for people intending to be active. Rehabil Psychol 2013;58(3):307-15. doi: 10.1037/a0032816 [published Online First: 2013/08/28]

36. Lachman ME, Lipsitz L, Lubben J, et al. When Adults Don't Exercise: Behavioral Strategies to Increase Physical Activity in Sedentary Middle-Aged and Older Adults. Innov Aging 2018;2(1):igy007. doi: 10.1093/geroni/igy007 [published Online First: 2018/07/14]

37. Ginis KA, Tomasone JR, Latimer-Cheung AE, et al. Developing physical activity interventions for adults with spinal cord injury. Part 1: a comparison of social cognitions across actors, intenders, and nonintenders. Rehabil Psychol 2013;58(3):299-306. doi: 10.1037/a0032815 [published Online First: 2013/08/28]

38. MacKay C, Lee L, Best K, et al. Developing a research agenda on exercise and physical activity for people with limb loss in Canada. Disabil Rehabil 2021:1-9. doi: 10.1080/09638288.2021.2003877 [published Online First: 2021/11/30]

39. Bouzas S, Molina AJ, Fernandez-Villa T, et al. Effects of exercise on the physical fitness and functionality of people with amputations: Systematic review and meta-analysis. Disabil Health J 2021;14(1):100976. doi: 10.1016/j.dhjo.2020.100976 [published Online First: 2020/08/21]

40. MacKay C LL, Mendelsohn S, Kobylansky A, Dilkas S, Devlin M, Mayo A, Hitzig SL. Exercise and Physical Activity Interventions for People with Lower Limb Amputations: A Scoping Review. KT Canada Scientific Conference. Virtual Conference, 2021.

41. Durstine JL, Painter P, Franklin BA, et al. Physical activity for the chronically ill and disabled. Sports Med 2000;30(3):207-19. doi: 10.2165/00007256-200030030-00005 [published Online First: 2000/09/22]

42. Christiansen CL, Miller MJ, Murray AM, et al. Behavior-Change Intervention Targeting Physical Function, Walking, and Disability After Dysvascular Amputation: A Randomized Controlled Pilot Trial. Arch Phys Med Rehabil 2018;99(11):2160-67. doi: 10.1016/j.apmr.2018.04.011 [published Online First: 2018/05/11]

43. Imam B, Miller WC, Finlayson H, et al. A randomized controlled trial to evaluate the feasibility of the Wii Fit for improving walking in older adults with lower limb amputation. Clin Rehabil 2017;31(1):82-92. doi: 10.1177/0269215515623601 [published Online First: 2016/01/02]

44. Pauley T, Devlin M, Madan-Sharma P. A single-blind, cross-over trial of hip abductor strength training to improve Timed Up & Go performance in patients with unilateral, transfemoral amputation. J Rehabil Med 2014;46(3):264-70. doi: 10.2340/16501977-1270 [published Online First: 2013/12/24]

45. Schafer ZA, Perry JL, Vanicek N. A personalised exercise programme for individuals with lower limb amputation reduces falls and improves gait biomechanics: A block randomised controlled trial. Gait Posture 2018;63:282-89. doi: 10.1016/j.gaitpost.2018.04.030 [published Online First: 2018/05/29]

46. Dupuis F, Ginis KAM, MacKay C, et al. "Do exercise programs improve fitness, mobility and functional capacity in adults with lower limb amputation? A systematic review on the type and minimal dose needed''. Arch Phys Med Rehabil 2023 doi: 10.1016/j.apmr.2023.10.011 [published Online First: 2023/11/06]

47. Christiansen CL, Miller MJ, Kline PW, et al. Biobehavioral Intervention Targeting Physical Activity Behavior Change for Older Veterans after Nontraumatic Amputation: A Randomized Controlled Trial. PM R 2020;12(10):957-66. doi: 10.1002/pmrj.12374 [published Online First: 2020/04/06]

48. Gourlan M, Bernard P, Bortolon C, et al. Efficacy of theory-based interventions to promote physical activity. A meta-analysis of randomised controlled trials. Health Psychology Review 2016;10(1):50-66. doi: 10.1080/17437199.2014.981777

49. McAuley E, Szabo A, Gothe N, et al. Self-efficacy: Implications for Physical Activity, Function, and Functional Limitations in Older Adults. Am J Lifestyle Med 2011;5(4) doi: 10.1177/1559827610392704 [published Online First: 2011/07/01]

50. Neupert SD, Lachman ME, Whitbourne SB. Exercise self-efficacy and control beliefs: effects on exercise behavior after an exercise intervention for older adults. Journal of aging and physical activity 2009;17(1):1-16. doi: 10.1123/japa.17.1.1

51. Bandura A. Self-Efficacy: The Exercise of Control. New York: WH Freeman 1997.

52. Ryan RM, Deci EL. Self-determination theory and the facilitation of intrinsic motivation, social development, and well-being. Am Psychol 2000;55(1):68-78. doi: 10.1037//0003-066x.55.1.68 [published Online First: 2001/06/08]

53. van Twillert S, Postema K, Geertzen JH, et al. Incorporating self-management in prosthetic rehabilitation: case report of an integrated knowledge-to-action process. Physical Therapy 2015;95(4):640-47.

54. Wegener ST, Mackenzie EJ, Ephraim P, et al. Self-management improves outcomes in persons with limb loss. Archives of Physical Medicine and Rehabilitation 2009;90(3):373-80.

55. Pepin M AK, Galen S. Physical activity in individuals with lower extremity amputations: a narrative review. Physical Therapy Reviews 2018;23(2):10.

56. Wezenberg D, Dekker R, van Dijk F, et al. Cardiorespiratory fitness and physical strain during prosthetic rehabilitation after lower limb amputation. Prosthet Orthot Int 2019;43(4):418-25. doi: 10.1177/0309364619838084 [published Online First: 2019/03/25]

57. Gutnick D, Reims K, Davis C, et al. Brief action planning to facilitate behavior change and support patient self-management. Journal of Clinical Outcomes Management 2014;21:17-29.

58. Veerabhadrappa P, Moran MD, Renninger MD, et al. Tracking Steps on Apple Watch at Different Walking Speeds. J Gen Intern Med 2018;33(6):795-96. doi: 10.1007/s11606-018-4332-y

59. Karinharju KS, Boughey AM, Tweedy SM, et al. Validity of the Apple Watch® for monitoring push counts in people using manual wheelchairs. The Journal of Spinal Cord Medicine 2021;44(2):212-20. doi: 10.1080/10790268.2019.1576444

60. Schembre SM, Liao Y, Robertson MC, et al. Just-in-Time Feedback in Diet and Physical Activity Interventions: Systematic Review and Practical Design Framework. J Med Internet Res 2018;20(3):e106. doi: 10.2196/jmir.8701 [published Online First: 2018/03/24]

61. Bassett DR, Troiano RP, McClain JJ, et al. Accelerometer-based physical activity: total volume per day and standardized measures. Med Sci Sports Exerc 2015;47(4):833-8. doi: 10.1249/mss.0000000000000468 [published Online First: 2014/08/08]

62. LaCroix AZ, Bellettiere J, Rillamas-Sun E, et al. Association of Light Physical Activity Measured by Accelerometry and Incidence of Coronary Heart Disease and Cardiovascular Disease in Older Women. JAMA Network Open 2019;2(3):e190419-e19. doi: 10.1001/jamanetworkopen.2019.0419

63. Saint-Maurice PF, Troiano RP, Matthews CE, et al. Moderate-to-Vigorous Physical Activity and All-Cause Mortality: Do Bouts Matter? J Am Heart Assoc 2018;7(6) doi: 10.1161/jaha.117.007678 [published Online First: 2018/03/24]

64. Ladlow P, Nightingale TE, McGuigan MP, et al. Impact of anatomical placement of an accelerometer on prediction of physical activity energy expenditure in lower-limb amputees. PLoS One 2017;12(10):e0185731. doi: 10.1371/journal.pone.0185731 [published Online First: 2017/10/06]

65. García-Massó X, Serra-Añó P, García-Raffi LM, et al. Validation of the use of Actigraph GT3X accelerometers to estimate energy expenditure in full time manual wheelchair users with spinal cord injury. Spinal Cord 2013;51(12):898-903. doi: 10.1038/sc.2013.85 [published Online First: 2013/09/04]

66. Resnick B, Jenkins LS. Testing the Reliability and Validity of the Self-Efficacy for Exercise Scale. Nursing Research 2000;49(3)

67. Woodgate J, Brawley LR. Self-efficacy for exercise in cardiac rehabilitation: review and recommendations. J Health Psychol 2008;13(3):366-87. doi: 10.1177/1359105307088141 [published Online First: 2008/04/19]

68. van Dijk SEM, Adriaanse MC, van der Zwaan L, et al. Measurement properties of depression questionnaires in patients with diabetes: a systematic review. Qual Life Res 2018;27(6):1415-30. doi: 10.1007/s11136-018-1782-y [published Online First: 2018/02/06]

69. Washburn RA, Zhu W, McAuley E, et al. The physical activity scale for individuals with physical disabilities: development and evaluation. Arch Phys Med Rehabil 2002;83(2):193-200. [published Online First: 2002/02/08]

70. van der Ploeg HP, Streppel KR, van der Beek AJ, et al. The Physical Activity Scale for Individuals with Physical Disabilities: test-retest reliability and comparison with an accelerometer. J Phys Act Health 2007;4(1):96-100. doi: 10.1123/jpah.4.1.96 [published Online First: 2007/05/10]

71. Martin Ginis KA, Phang SH, Latimer AE, et al. Reliability and validity tests of the leisure time physical activity questionnaire for people with spinal cord injury. Arch Phys Med Rehabil 2012;93(4):677-82. doi: 10.1016/j.apmr.2011.11.005 [published Online First: 2012/02/18]

72. Miller WC, Deathe AB, Speechley M. Psychometric properties of the Activities-specific Balance Confidence scale among individuals with a lower-limb amputation11No commercial party having a direct financial interest in the results of the research supporting this article has or will confer a benefit upon the author(s) or upon any organization with which the author(s) is/are associated. Archives of Physical Medicine and Rehabilitation 2003;84(5):656-61. doi: <https://doi.org/10.1016/S0003-9993(02)04807-4>

73. Franchignoni F, Ferriero G, Giordano A, et al. The minimal clinically-important difference of the Prosthesis Evaluation Questionnaire - Mobility Scale in subjects undergoing lower limb prosthetic rehabilitation training. Eur J Phys Rehabil Med 2020;56(1):82-87. doi: 10.23736/S1973-9087.19.05799-X [published Online First: 2019/09/07]

74. Franchignoni F, Giordano A, Ferriero G, et al. Rasch analysis of the Locomotor Capabilities Index-5 in people with lower limb amputation. Prosthet Orthot Int 2007;31(4):394-404. doi: 10.1080/03093640701253952 [published Online First: 2007/12/01]

75. Jakobsson U. Using the 12-item Short Form health survey (SF-12) to measure quality of life among older people. Aging Clin Exp Res 2007;19(6):457-64. doi: 10.1007/BF03324731 [published Online First: 2008/01/04]

76. Miller WC, Deathe AB, Harris J. Measurement properties of the Frenchay Activities Index among individuals with a lower limb amputation. Clin Rehabil 2004;18(4):414-22. doi: 10.1191/0269215504cr728oa [published Online First: 2004/06/08]

77. Norvell DC, Williams RM, Turner AP, et al. The development and validation of a novel outcome measure to quantify mobility in the dysvascular lower extremity amputee: the amputee single item mobility measure. Clin Rehabil 2016;30(9):878-89. doi: 10.1177/0269215516644308 [published Online First: 2016/08/09]

78. Nasreddine ZS, Phillips NA, Bedirian V, et al. The Montreal Cognitive Assessment, MoCA: a brief screening tool for mild cognitive impairment. J Am Geriatr Soc 2005;53(4):695-9. doi: 10.1111/j.1532-5415.2005.53221.x [published Online First: 2005/04/09]

79. Sandelowski M. Whatever happened to qualitative description? Res Nurs Health 2000;23(4):334-40. doi: 10.1002/1098-240x(200008)23:4<334::aid-nur9>3.0.co;2-g [published Online First: 2000/08/15]

80. Denzin N, Lincoln,Y. Handbook of Qualitative Research: Thousand Islands, California: Sage Publications Inc 2000.

81. Miller MJ, Jones J, Anderson CB, et al. Factors influencing participation in physical activity after dysvascular amputation: a qualitative meta-synthesis. Disabil Rehabil 2018:1-10. doi: 10.1080/09638288.2018.1492031 [published Online First: 2018/09/29]

84. Whitehead AL, Julious SA, Cooper CL, et al. Estimating the sample size for a pilot randomised trial to minimise the overall trial sample size for the external pilot and main trial for a continuous outcome variable. Stat Methods Med Res 2016;25(3):1057-73. doi: 10.1177/0962280215588241 [published Online First: 2015/06/21]

85. Langford J, Dillon MP, Granger CL, et al. Physical activity participation amongst individuals with lower limb amputation. Disabil Rehabil 2018:1-8. doi: 10.1080/09638288.2017.1422031 [published Online First: 2018/01/06]

86. Mellor, K., Albury, C., Dutton, S. J., Eldridge, S., & Hopewell, S. (2023). Recommendations for progression criteria during external randomised pilot trial design, conduct, analysis and reporting. Pilot and feasibility studies, 9(1), 59. https://doi.org/10.1186/s40814-023-01291-5
